# Tetrandrine ameliorated atherosclerosis in vitamin D3/high cholesterol diet-challenged rats via modulation of miR-34a and Wnt5a/Ror2/ABCA1/NF-kB trajectory

**DOI:** 10.1038/s41598-024-70872-y

**Published:** 2024-09-12

**Authors:** Yasmin El Zouka, Eman Sheta, Mona Abdelrazek Salama, Eman Selima, Rowaida Refaat, Sherihan Salaheldin Abdelhamid Ibrahim

**Affiliations:** 1 Department of Pharmacology, Faculty of Pharmacy, Arab Academy for Science and Technology and Maritime Transport, Alexandria, Egypt; 2 Department of Pathology, Faculty of Medicine, Alexandria University, Alexandria, Egypt; 3 Department of Pharmacology, Medical Research Institute, Alexandria University, Alexandria, Egypt; 4 Department of Pharmacology and Therapeutics, Faculty of Pharmacy, Pharos University in Alexandria, Alexandria, Egypt

**Keywords:** HCD (high cholesterol diet), AS (atherosclerosis), Tetrandrine, Wnt5a/Ror2/ABCA1/NF-κB pathway, miR-34, Cardiology, Diseases, Health care, Medical research, Molecular medicine, Pathogenesis

## Abstract

Atherosclerosis (AS) is a major cause of cardiovascular diseases that may lead to mortality. This study aimed to evaluate the therapeutic potential of tetrandrine in high cholesterol diet (HCD)-induced atherosclerosis, in rats, via modulation of miR-34a, as well as, Wnt5a/Ror2/ABCA1/NF-κB pathway and to compare its efficacy with atorvastatin. Induction of AS, in male rats, was done via IP administration of vitamin D3 (70 U/Kg for 3 days) together with HCD. At the end of the 9th week, rats were treated with atorvastatin at a dose of 20 mg/kg, and tetrandrine at different doses of (18.75, and 31.25 mg/kg) for 22 days. Serum inflammatory cytokines and lipid profile, liver oxidative stress parameters, and aortic tissue Wnt5a, Ror2, ABCA1, NF-κB, miR-34a levels were assessed in all experimental groups. Histopathological and Immunohistochemical assessments of aortic tissue sections were done. Results showed that tetrandrine treatment reverted the inflammatory and oxidative stress state together with reducing the serum lipids via modulating miR-34a, and Wnt5a/Ror2/ABCA1/NF-κB pathway. Moreover, it reverted the histopathological abnormalities observed in AS rats. Tetrandrine beneficial effects, in both doses, were comparable to that of atorvastatin, in most of the discussed parameters. These findings praise tetrandrine as a promising agent for management of atherosclerosis.

## Introduction

Atherosclerosis (AS) is one of the critical health issues that increases the risk of cardiovascular diseases (CVDs) and stroke. It is characterized by the accumulation of cholesterol or lipoproteins such as low-density lipoproteins (LDL), fibrous tissue, and calcification within the arteries. These events subsequently lead to the narrowing of blood vessels and activation of inflammatory pathways leading to atheroma plaque formation^1^.

Atherosclerosis occurs as the initial stage of vascular endothelial dysfunction that causes oxidative stress and inflammation in the artery walls, which exacerbates the dysfunction itself^2^. The imbalance between anti-oxidant and pro-oxidant mediators together with inflammation forms a vicious cycle that plays an important role in the pathophysiology and exacerbation of atherosclerosis^3^. Searching for a new treatment that could halt both inflammation and oxidative stress would be a promising option to slow the progression of atherosclerosis and avoid cardiovascular problems^2^.

Wingless (WNT) are a group of lipid-modified glycoproteins that play an important physiological role in cell proliferation, migration, and differentiation^4^. Perturbations in Wnt5a signaling have been reported in various inflammatory diseases such as rheumatoid arthritis, atherosclerosis and psoriasis^4^. Furthermore, it has been found that Wnt5a is highly expressed in the atherosclerotic plaque and the serum of atherosclerotic patients, this highlights its pathological role in atherosclerosis^5^.

Also, it has been shown that Wnt5a assembles to receptor tyrosine kinase-like orphan receptor 2 (Ror2), that are highly expressed in foam cells of the atherosclerotic plaque^6^. Moreover, it was highlighted that binding of Wnt5a to Ror2 could inhibit Adenosine triphosphate (ATP)-binding cassette transporter A1 (ABCA1), which is a transporter that can efflux excess cholesterol from various cells^7,8^. Also, it has been demonstrated previously that miR-34a plays a crucial role in inhibiting ABCA1^9^. This subsequently would lead to cholesterol accumulation and activation of nuclear factor kappa B (NF-κB) that finally leads to increment in the inflammatory cytokines level such as tumor necrosis factor-alpha (TNF-α) and interleukin-6 (IL-6)^8,10^. We concluded from all of the previous findings that miR-34a together with Wnt5a/Ror2/NF-κB trajectory could have a great role in the pathophysiology of atherosclerosis.

Tetrandrine is considered one of the alkaloids extracted from *Stephania tetrandrae* S. that possess anti-inflammatory, anti-oxidant, anti-cancer, and anti-arrhythmic actions^11–13^. Previously, the anti-inflammatory effect of tetrandrine was shown in rheumatoid arthritis induced rat model and the anti-inflammatory effect of high-dose tetrandrine was similar to that of methotrexate^14^. Furthermore, a prior study showed the ability of this alkaloid to suppress the Wnt signaling trigger in Human Colorectal Cancer^15,16^.

Many adverse effects are associated with the use of conventional treatments for atherosclerosis (ezetimibe, bile acid binding resins, statins, and others)^17^. Based on the previously provided data, we deduced that discovering an alternative natural treatment for atherosclerosis that combines anti-inflammatory and anti-oxidant properties may represent a novel avenue for investigation. Therefore, the current study's goal was to evaluate tetrandrine's therapeutic potential in a rat atherosclerosis model by modifying the miR-34a and Wnt5a/Ror2/NF-κB cue.

## Materials and methods

### Animals

Fifty-six Male Sprague–Dawley rats were purchased from the animal house, faculty of pharmacy, Pharos University in Alexandria. Their weight ranged from 150 to 175 gm. Rats were kept under normal laboratory conditions (temperature 24 ± 2°C, air humidity 50 ± 15%, 12 h light–dark cycle) and had free access to food and drinking water for 7 days for acclimatization. All experimental procedures were performed according to the Guide for the Care and Use of Laboratory Animals (NIH), the Animal Research: Reporting of in vivo Experiments (ARRIVE) guidelines, and approved by the “Alexandria University-Institutional Animal Care and Use Committee (ALEXU-IACUC), Medical research institute in Alexandria” (AU-0122252211).

### Diet induced atherosclerosis in rats

Atherosclerosis was induced in 40 rats by administering vitamin D3 IP (70 U/kg) continuously for 3 days. This was done together by feeding the animals with high cholesterol diet (HCD), containing the following ingredients as normal diet (80.3%), animal oil (11%), cholesterol (4.5%), sodium cholate (1.5%), propylthiouracil (0.7%) and refined sugar (4%) till the (9th week)^18^.

### Assessment of atherosclerosis induction

At the end of the 9th week, induction of atherosclerosis was confirmed by determination of the serum lipid profile parameters; total triglyceride (TG), total cholesterol (TC), LDL, and high-density lipoprotein (HDL), and by histopathological examination of the aortic vessels of 16 selected rats; 8 from the normal control and 8 from the atherosclerotic groups^18^.

### Experimental groups

**Fig. 1:**
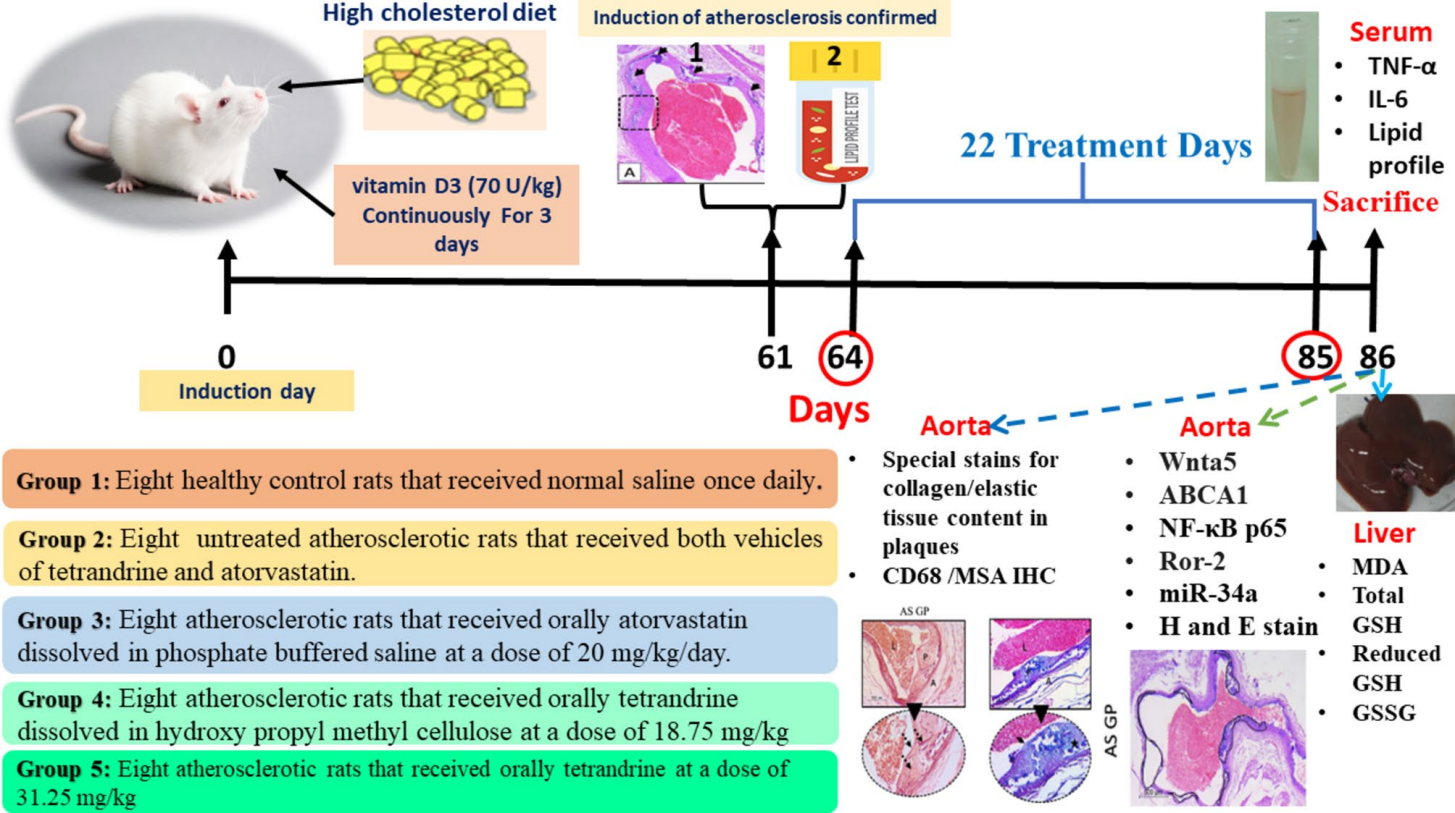
Experimental timeline for drug administration.

Figure 1 illustrates the experimental timeline for drug administration. The animals were randomly allocated into 5 groups as follows: Group 1 consisted of eight healthy control rats that received normal saline once daily, orally. Group 2 consisted of eight atherosclerotic rats that received both vehicles of atorvastatin and tetrandrine. Group 3 consisted of eight atherosclerotic rats that received orally atorvastatin (Egyptian Int. Pharmaceutical Industries CO.), dissolved in phosphate-buffered saline at a dose of 20 mg/kg/day^19^. Group 4 consisted of eight atherosclerotic rats that received orally tetrandrine, dissolved in hydroxy propyl methyl cellulose, (Cat# T2695, Sigma-Aldrich, USA) at a dose of 18.75 mg/kg^14^. Group 5 consisted of eight atherosclerotic rats that received orally tetrandrine at a dose of 31.25 mg/kg^14^. Starting from the 10th week, treatments were given to the animals in different experimental groups daily for 22 days^14^.

### Serum parameters

At the end of the study, animals were anesthetized with ketamine 100 mg/kg and xylazine 10 mg/kg that were given intraperitoneally. Blood samples were collected via cardiac puncture,and then centrifuged at 3000 rpm for 15 min to obtain serum samples.

#### Determination of serum inflammatory cytokines level

The serum obtained was used for the determination of TNF‐α (Cat# ab236712, Abcam, USA), and IL‐6 (Cat# BMS625, Invitrogen, USA) levels by using ELISA kits according to the manufacturer’s instructions.

#### Lipid profile

The TG, TC, and HDL were measured using standard enzymatic methods with an automated analyzer (Hitachi 912, Hitachi Ltd., Tokyo, Japan). HDL cholesterol was measured after phosphotungstic acid and magnesium precipitation. LDL cholesterol concentration was calculated using the Friedwald formula^20^. The atherogenic index (AI) was calculated by using the following formula: Atherogenic index = (TC-HDL-C)/HDL-C^21^.

### Tissue parameters

At the end of the study, the total aorta and liver were excised, washed with saline and then dried on filter paper. Excised Aortic vessel from each rat of different experimental groups was divided into 4 parts. The liver tissues and 3 parts of the excised aortic tissues form each rat were then preserved at − 80 °C for future investigation, while the remainder of the aortic tissues were fixed in 10% buffered formalin for 24 h. On the next day, they were serially sectioned and placed (well-oriented in the face) on plastic cassettes. Finally, they were embedded in paraffin blocks for histological evaluation.

#### Colorimetric assay

In the liver, oxidative stress parameters such as malondialdehyde (MDA) (Cat. no. MBS741034, My BioSource, USA), reduced GSH (Cat. no. MAK364, Sigma-Aldrich, USA), Total GSH and GSSG (Cat. no. E-BC-K097-S, Elabscience, USA) were determined by using colorimetric assay kits according to the manufacturer’s instructions.

#### ELISA assay

In the aorta, Wnta5 protein level (Cat. no. MBS2883334, My BioSource, USA) and ABCA1 protein level (Cat. no. MBS2512328, My BioSource, USA) were determined by using ELISA assay kits according to the manufacturer’s instructions.

#### Western blot analysis

Cytoplasmic and nuclear NF-κB p65 as well as Ror2 were determined in the aortic tissues by using western blot analysis technique. Samples of aorta were obtained from each experimental group were homogenized and lysed with RIPA lysis buffer PL005 (Bio BASIC INC, Marhham Ontario L3R 8T4, Canada). Lysates were centrifuged at ~ 16,000 × g for 30 min at 4 °C. The supernatant was transferred to new tubes. A nuclear and cytoplasmic extraction kit (Thermo Scientific, USA) was used to separate nuclear and cytoplasmic proteins followed by protein concentration determination analysis by using Bradford protein assay kit (SK3041, BIO BASIC INC. Markham Ontario L3R 8T4 Canada). The proteins were separated by 10% sodium dodecyl sulfate–polyacrylamide gels (SDS-PAGE) and transferred to a polyvinylidene fluoride membrane (PVDF). Furthermore, the membranes were blocked for 2 h with 5% skim milk in TBST buffer and probed with primary antibodies as follows: NF-κB p65 (Cat. no. ab16502; 1:2000); Ror2 (Cat. no. sc-374174; 1:500), histone (H3) ( Cat. no. ab24834; 1:1000) and β-actin (Cat. no. sc-47778; 1:1000) followed by secondary horseradish peroxidase-conjugated antibody. The gray density of immunolabeled proteins was assessed after treatment with ECL substrate. Target nuclear protein levels were standardized against H3, while target cytoplasmic protein levels were balanced against β-actin.

#### Reverse transcription-polymerase chain reaction (RT-PCR)

In the aortic tissues, The MagMAX mirVana Total RNA Isolation Kit (Cat. No. A27828, Thermofisher Scientific, USA) was used to isolate the total RNA. Nanodrop® spectrophotometer was used to measure the absorbance of isolated miRNA at 260 nm for quantification of isolated miRNA. In the reverse transcription (RT) step, cDNA was reverse transcribed from miRNA samples using specific microRNA primers, Table 1, from the TaqMan® MicroRNA Assays and reagents from the TaqMan® MicroRNA Reverse Transcription Kit. The thermal cycler lead was heated at 112 °C to prevent loss of sample, then the temperature was held for 30 min at 16°C, then for 30 min at 42 °C and finally for 5 min at 85 °C. The reaction volume was set to 15.0 µL. In the PCR step, PCR products were amplified from cDNA samples using the TaqMan® MicroRNA Assay together with the TaqMan^®^ Universal PCR Master Mix. Amplification of cDNA was carried out on step-one real-time PCR system (Applied Biosystems). It was held at 50°C for 2 min with an initial step of enzyme activation at 94 °C for 10 min, followed by 40 cycles of denaturation at 95 °C for 15 s, annealing and extension at 60 °C for 60 s. The results were analyzed by relative quantification (Rq); It describes the change in the expression of the target gene relative to the reference group such as healthy control using 2 − ∆∆ct Method. The ∆∆CT is calculated by subtracting ∆CT of the test sample from a control. Fold change (FC) is calculated by raising 2 to the power of the negative∆∆CT value, with the following equations: ∆CT = CT (assessed gene) − CT (reference gene), ∆∆CT = ∆CT (sample) − ∆CT (internal control gene), and FC (or Rq) = 2 − ∆∆CT. Using the 2 − ∆∆CT method, the data was presented as the fold change in miRNA expression normalized to an endogenous control (U6) and relative to the healthy controls.

**Table 1:** Primer sequence.

Gene	
miR-34a	F:5′ “TGGCAGTGTCTTAGCTGGTT”3′
	R: 5′ “ GAACATGTCTGCGTATCTC”3′
miR-U6	F: 5′ “AACGCTTCACGAATTTGCGT”3′
	R: 5′ “CTCGCTTCGGCAGCACA”3′

### H&E staining to assess histopathologic changes in aortic walls

Using a rotatory semi-automated microtome (SLEE, series 5062, Germany), five microns thick sections of aorta embedded in paraffin blocks were cut and placed on glass slides. They were stained by hematoxylin and eosin (H&E) to be examined by light microscopy (Olympus, CX 23 R). The whole aorta was assessed for the areas of atheromas. Using a digital camera, different photos of the aorta were taken at × 40 power. Using image analysis software (Image J), the whole vessel area was measured by manually calculating the area of the wall without the inclusion of the lumen or adventitia, then the area of atheromas (lesional area) was manually traced and measured. The ratio of lesional area out of the whole vessel wall area (represented as a percentage) was then calculated^22^. At × 400 power, atheromas were photographed to measure the intimal and media thickness in microns through the thickest part of the atheroma^23^.

### Special stains for collagen and elastic tissue content in atheroma:

Two serial sections of Paraffin blocks were cut on glass slides to be stained by Orcein staining kit for elastic fibers assessment (Cat# HBK-IFU, ScyTech laboratories, USA) and Trichrome Stain Kit (Modified Masson's) (Cat# TRM-IFU, ScyTech laboratories, USA) for fibrous tissue assessment in studied groups. At × 200 power, different photos of the aortic wall and areas of atheromatous were captured using a digital microscope camera. Using image J software, areas of atheroma were manually selected and these photos were changed into 8-bit greyscale. Segmentation of color was done and color of interest was chosen to calculate the percentage of elastic or fibrous tissue within the atheroma according to this equation (collagen or elastic tissue area/atheroma area × 100% in × 200 power field)^24^.

### Immunohistochemical staining to assess macrophage and smooth muscle content in plaques

Two serial five microns thick sections were cut from paraffin blocks. They were placed on positively charged slides to be deparaffinized and hydrated. Incubation with hydrogen peroxide for 15 min was done to block endogenous peroxidase. Antigen retrieval was performed by heating with citrate buffer at 90 degrees for 10 min. Slides were then incubated overnight with primary antibodies by anti-CD68 (Cat# MA5-13324, Invitrogen, USA) as a marker of macrophages and anti-muscle specific acting (MSA) (Cat# MSA-594-L-U, Leica biosystems, Germany) as a marker of smooth muscles. On the following day labeled polymer horseradish peroxidase (HRP Polymer universal kit) was used (Conjugate, Invitrogen, USA). Colour reaction was developed by adding DAB as a chromogen and slides were then counterstained by hematoxylins and cover slipped. Examination under light microscopy was done to assess the expression of both markers. In both markers, positivity was seen as brown cytoplasmic staining. Nuclei were stained blue. The assessment was done in atheromas seen in the media with avoidance of adventitia. The brown staining positive area was calculated as a percentage of total area examined^25^. Immunohistochemical staining and analysis were done in image analysis unit (pathology laboratory, faculty of medicine, Alexandria University).

### Statistical analysis

The software employed was GraphPad Prism Version 8. Values are expressed as mean ± standard deviation (mean of 8 values/group). One-way analysis of variance (ANOVA) was used followed by Tukey as a post hoc test for multiple comparisons. Moreover, in some parameters that were wanted to compare 2 groups, a Paired t test was used. The *P* value less than 0.05 was considered to be statistically significant.

## Results

### Assessment of atherosclerosis induction:

#### Serum lipid profile

**Fig. 2:**
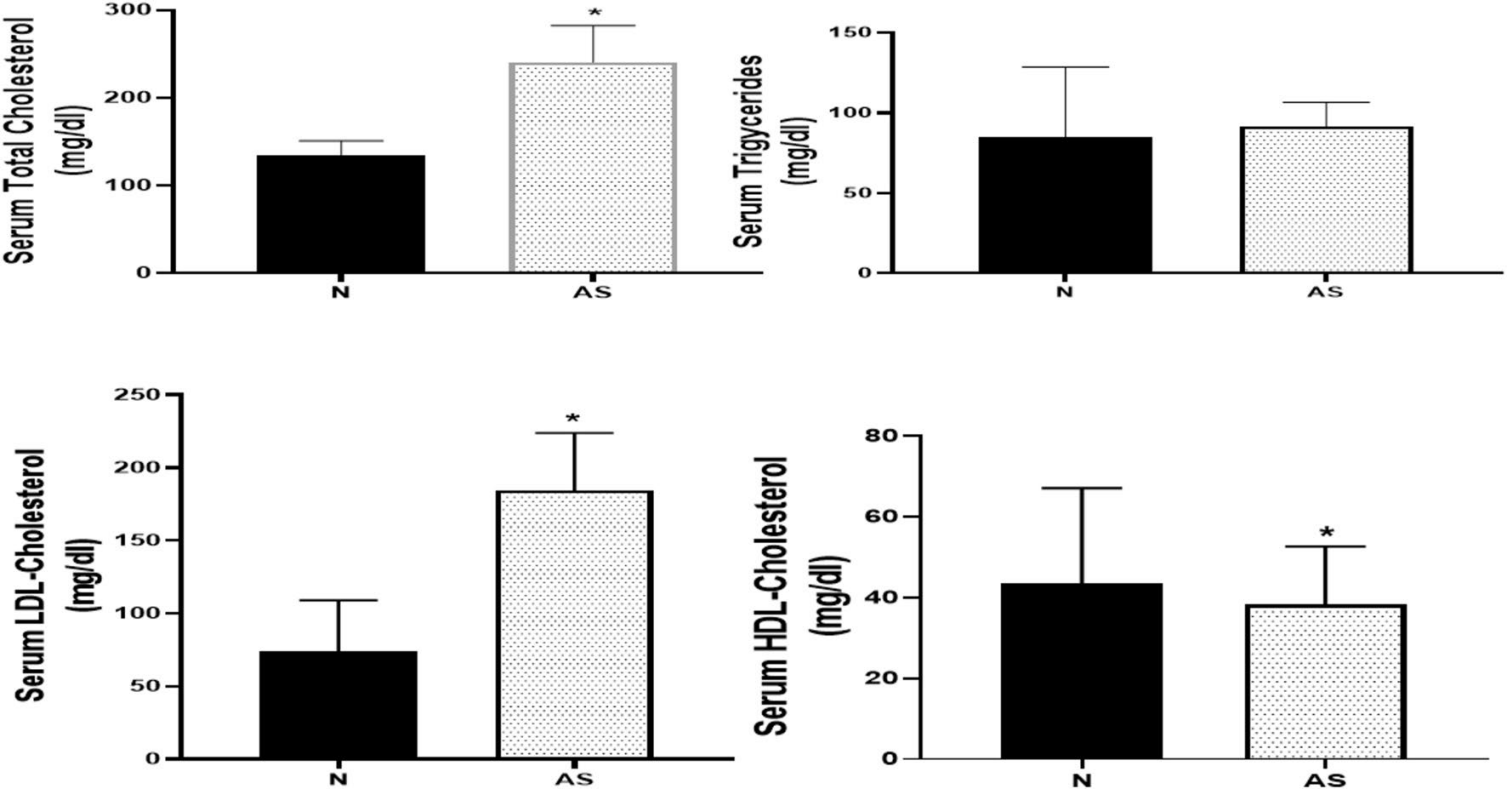
Change in the serum lipid profile of rats after 9 weeks of feeding on high-cholesterol diet (HCD) together with vitamin D3 administration (70 U/kg) continuously for 3 days as compared to normal rats. Data represented as mean ± SD of 8 rats. Paired t test was used, *P* value less than 0.05 was considered to be statistically significant. *is significantly different from N. N: normal control group, AS: Atherosclerotic group.

Figure 2 illustrates the change in the serum lipid profile of rats after 9 weeks of feeding on HCD together with vitamin D3 administration (70 U/kg) for 3 days. Our results showed a significant increase in the serum cholesterol and LDL levels reaching (240.8 ± 41.36, and 184.2 ± 39.62 mg/dl, respectively) as well as a significant decrease in the serum HDL level reaching (43.47 ± 23.71 mg/dl) in AS rats as compared to normal rats (134.4 ± 16.56, 73.88 ± 35.03, and 38.26 ± 14.45 mg/dl, respectively). Moreover, there was no significant difference between AS and normal groups in the serum TG level (91.45 ± 14.99, and 85.02 ± 43.48 mg/dl, respectively).

#### Histopathological assessments

**Fig. 3:**
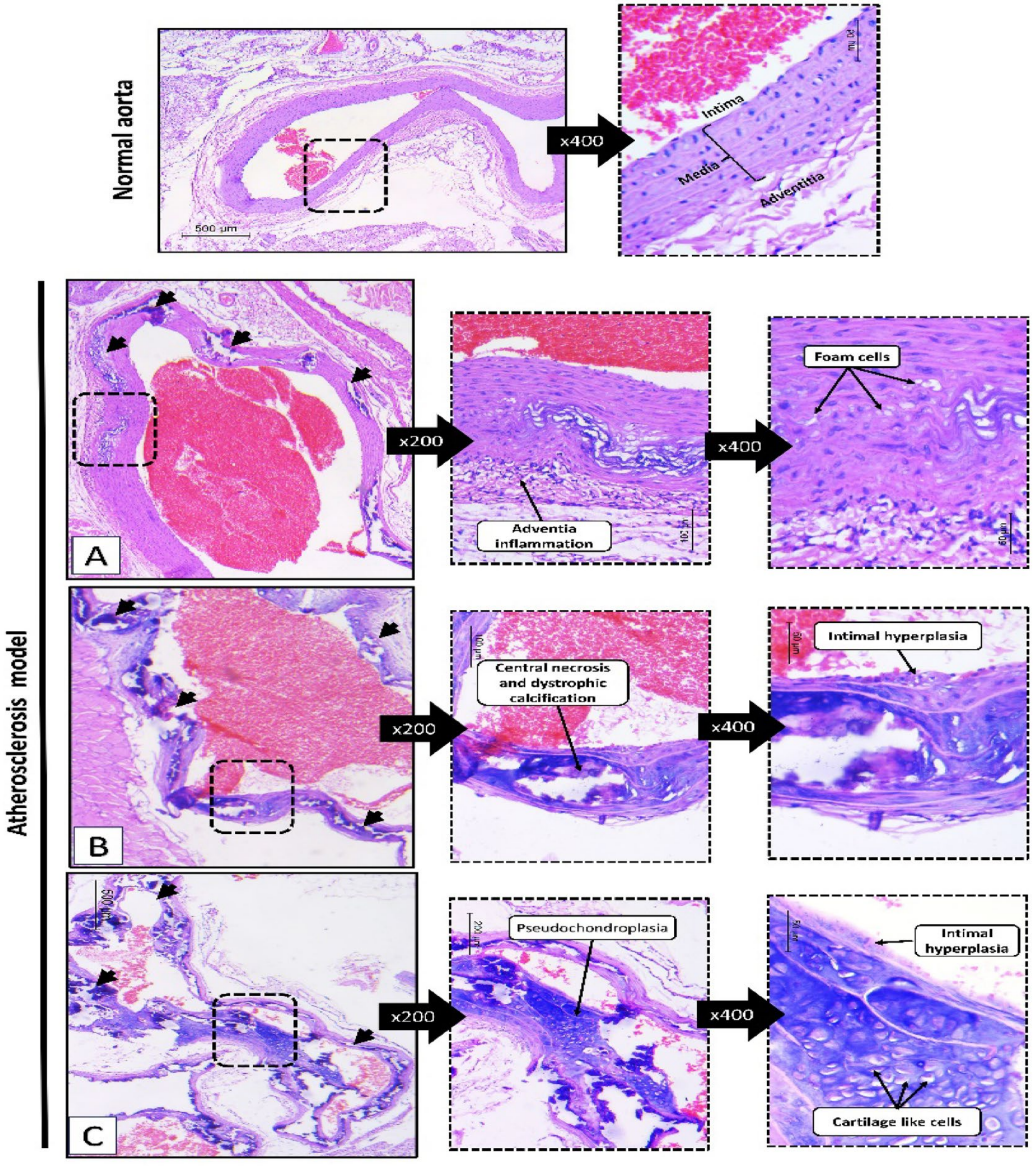
The histopathological changes of the rats' aorta after 9 weeks of feeding on high-cholesterol diet (HCD) together with vitamin D3 administration (70 U/kg) continuously for 3 days as compared to normal rats. Normal group shows normal vessel wall formed of three layers (intima, media and adventitia). (**A**–**C**) The atherosclerosis group shows evident atheromas (black arrows) with classic pathologic changes. (**A**) Foam cells infiltration and adventitial inflammation. (**B**) Dystrophic calcifications and intimal hyperplasia. (**C**) Pseudochondroplasia. (H&E, low power × 40, higher powers (× 200 and × 400).

Figure 3, in the normal rats, the dissected aorta showed normal histology. The vessel wall was formed of three layers. The first layer facing the lumen was the intima. It was formed of a single layer of flat endothelial cells. It was resting on tunica media which is the thickest layer. It was formed of smooth muscle cells arranged in an ordinal fashion. Lastly, the adventitia was formed of loose connective tissue showing few small thin capillaries.

In AS group, the aorta showed yellow streaks by naked eye appearance. On histologic level, the wall showed multiple atheromatous plaques. Some of them were seen as subintimal collection of foamy cells. They were seen dissecting in between media smooth muscle cells. The adventitia was inflamed. In other areas, atheromas were more developed. They occupied most of the vessel wall. They showed central necrosis and dense dystrophic calcification. The intimal over the atheromas were hyperplastic and thick. In some foci, it was totally ulcerated. In some atheromas, pseudochondroplasia was well seen. It was formed of cartilage-like cells within a homogenous background.

### Serum parameters

#### Serum inflammatory cytokines level

**Fig. 4:**
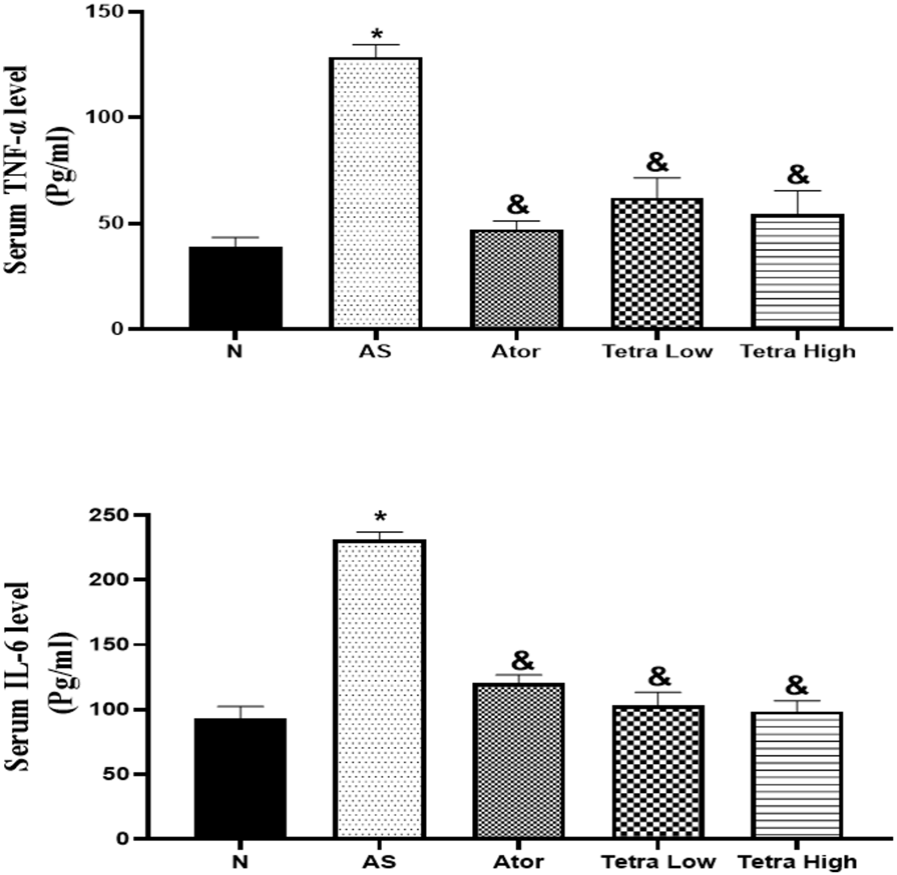
Change in the serum inflammatory cytokines in AS rats induced by vitamin D3/high cholesterol diet (HCD) after administration of atorvastatin at a dose of 20 mg/kg/day, or tetrandrine at low or high dose for 22 days. Data represented as mean ± SD of 8 rats. One way ANOVA test was used followed by Tukey as post hoc test for multiple comparisons. *is significantly different from N. & is significantly different from AS. N: normal control group, AS: Atherosclerotic group, Ator: atorvastatin treated group, Tetra low: tetrandrine treated group at a dose of 18.75 mg/kg, and Tetra high: tetrandrine treated group at a dose of 31.25 mg/kg.

Figure 4, the serum TNF-α, and IL-6 levels were significantly elevated by 3.30, and 2.48 folds, respectively in the AS rats as compared to normal ones. While treatment with atorvastatin at the aforementioned dose significantly declined the serum TNF-α, and IL-6 levels by 63.31, and 47.84%, respectively as compared to AS rats. Moreover, treatment with tetrandrine at low and high doses significantly mitigated the serum TNF-α level by (51.69, and 57.60%, respectively). The serum level of IL-6 was declined by (55.27 and 57.36%, respectively) in the rats treated with low or high dose of tetrandrine as compared to AS rats. There was no significant difference between experimental groups treated with low and high dose of tetrandrine. These results showed the anti-inflammatory effect of tetrandrine.

#### Serum lipid profile

**Fig. 5:**
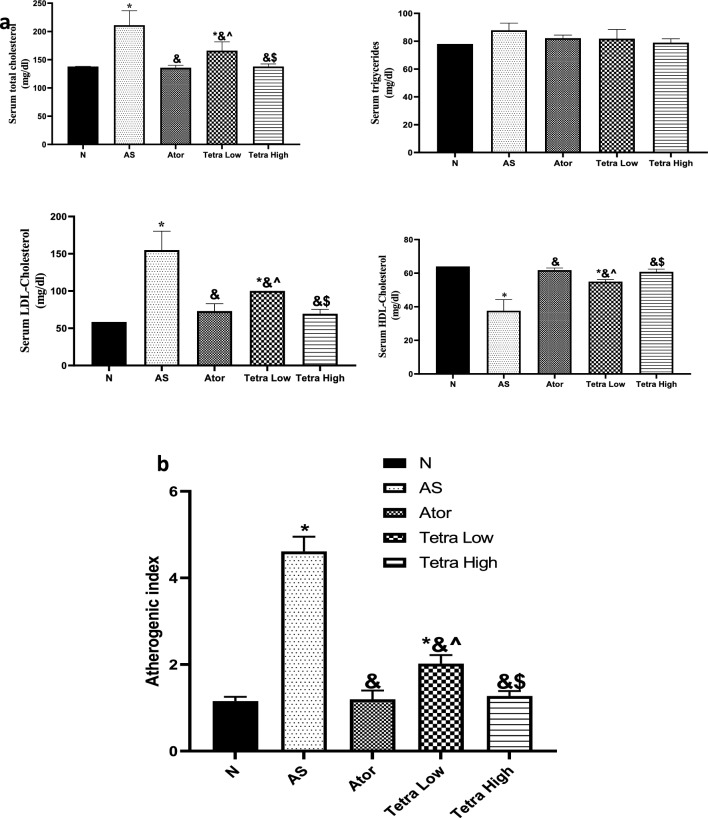
(**a**) Change in the serum lipid profile of AS rats induced by vitamin D3/high cholesterol diet (HCD) after administration of atorvastatin at a dose of 20 mg/kg/day, or tetrandrine at low or high dose for 22 days. Data represented as mean ± SD of 8 rats. One way ANOVA test was used followed by Tukey as post hoc test for multiple comparisons. *is significantly different from N. & is significantly different from AS. ^ is significantly different from Ator. $ is significantly different from Tetra low. N: normal control group, AS: Atherosclerotic group, Ator: atorvastatin treated group, Tetra low: tetrandrine treated group at a dose of 18.75 mg/kg, and Tetra high: tetrandrine treated group at a dose of 31.25 mg/kg. (**b**) Bar chart representing atherogenic index calculated as follows: Atherogenic index = (TC-HDL-C)/HDL-C.

Figure 5 shows that the serum cholesterol, and LDL levels in AS rats were significantly elevated by 1.53, and 2.65 folds as compared to normal ones. There was no significant difference between as normal and AS groups regarding serum TG level. However, the serum HDL level was significantly decreased by 41.25% in AS rats as compared to normal.

The serum cholesterol and LDL levels were significantly mitigated by atorvastatin treatment by 35.64, and 52.82%, respectively. Tetrandrine low dose mitigated their levels by 21.32, and 35.44%, respectively. While tetrandrine in high dose declined their levels by 34.59, and 55.25%, respectively. There was no significant difference between all treated groups regarding the serum TG level. The serum HDL level was elevated by 1.64, 1.46, and 1.62 folds in rats treated with atorvastatin or tetrandrine low or high dose, respectively.

The atherogenic index was significantly increased in the AS group reaching (4.61) as compared to the normal group (1.15). treatment with atorvastatin and tetrandrine at low or high dose reduced the atherogenic index reaching (1.20, 2.02, 1.27), respectively. These findings demonstrate tetrandrine's potential as an anti-hyperlipidemic agent.

### Tissue parameters

#### Oxidative stress parameters

**Fig. 6:**
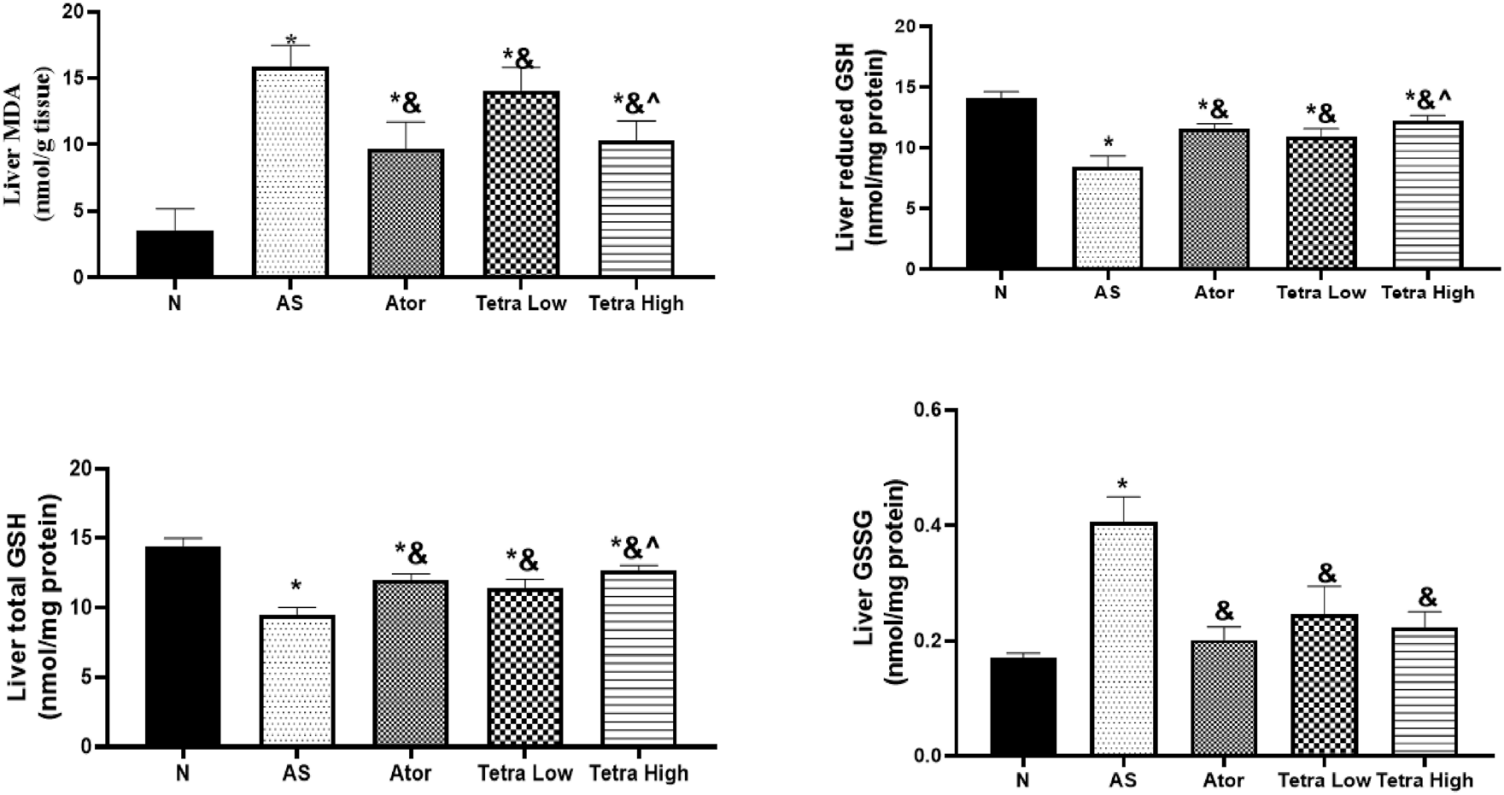
Change in the liver oxidative stress parameters in AS rats induced by vitamin D3/high cholesterol diet (HCD) after administration of atorvastatin at a dose of 20 mg/kg/day,or tetrandrine at low or high dose for 22 days. Data represented as mean ± SD of 8 rats. One way ANOVA test was used followed by Tukey as post hoc test for multiple comparisons. *is significantly different from N. & is significantly different from AS. ^ significantly different from Terta low. N: normal control group, AS: Atherosclerotic group, Ator: atorvastatin treated group, Tetra low: tetrandrine treated group at a dose of 18.75 mg/kg, and Tetra high: tetrandrine treated group at a dose of 31.25 mg/kg.

Figure 6 shows the change in the oxidative stress parameters in AS rats at the end of the study after administration of atorvastatin at a dose of 20 mg/kg/day, or tetrandrine at low dose of (18.75 mg/kg) or high dose (31.25 mg/kg) for 22 days as compared to AS untreated rats. Firstly, there was a significant increase in the liver MDA level in AS rats (15.82 ± 1.66 nmol/gm tissue) as compared to healthy rats (3.52 ± 1.67 nmol/gm tissue). Then, all drug treatments given significantly declined the liver MDA level reaching (9.7 ± 1.99, 14.03 ± 1.77, 10.30 ± 1.50 nmol/gm tissue), respectively.

Furthermore, the liver total GSH, and reduced GSH levels were significantly reduced in AS rats reaching (9.48 ± 0.54, and 8.42 ± 0.94 nmol/mg protein, respectively) as compared to healthy rats reaching (14.39 ± 0.62, 14.05 ± 0.61 nmol/mg protein, respectively). Atorvastatin treatment significantly elevated the liver total GSH and reduced GSH reaching (12.01 ± 0.42, and 11.61 ± 0.39 nmol/mg protein, respectively). Also, tetrandrine low dose significantly their levels reaching (11.41 ± 0.64, and 10.92 ± 0.69 nmol/mg protein, respectively). The high dose of tetrandrine significantly elevated their levels reaching (12.70 ± 0.36, and 12.26 ± 0.41 nmol/mg protein, respectively).

Moreover, the liver GSSG was increased in the AS rats (0.4 ± 0.04 nmol/mg protein) as compared to normal (0.17 ± 0.1 nmol/mg protein). All treatments administered declined GSSG liver level (0.20 ± 0.02, 0.25 ± 0.05, 0.22 ± 0.03 nmol/mg protein, respectively). This highlighted the anti-oxidant properties of tetrandrine.

#### Wnt5a and ABCA1 aortic tissue levels

**Fig. 7:**
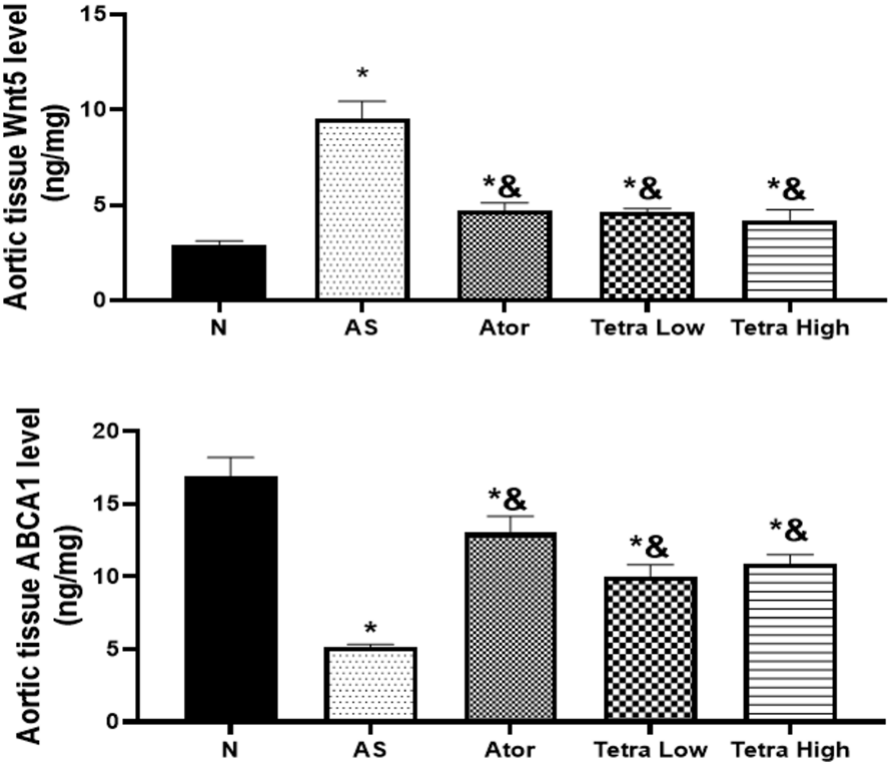
Change in the aortic tissue Wnt5a and ABCA1 levels in AS rats vitamin D3/induced by high cholesterol diet (HCD) after administration of atorvastatin at a dose of 20 mg/kg/day, or tetrandrine at low or high dose for 22 days. Data represented as mean ± SD of 8 rats. *is significantly different from N. & is significantly different from AS. N: normal control group, AS: Atherosclerotic group, Ator: atorvastatin treated group, Tetra low: tetrandrine treated group at a dose of 18.75 mg/kg, and Tetra high: tetrandrine treated group at a dose of 31.25 mg/kg.

Figure 7, there was a significant increase in the aortic tissue Wnt5a level (9.5 ± 0.94 ng/mg) and a decline in the ABCA1 level (5.16 ± 0.17 ng/mg) in the AS group as compared to the normal group (2.89 ± 0.23, 16.90 ± 1.31ng/mg, respectively). Atorvastatin or tetrandrine at the tested doses decreased the aortic tissue Wnt5 level in AS rats reaching (4.68 ± 0.44, 4.65 ± 0.17, 4.21 ± 0.54 ng/mg). Moreover, they elevated the aortic level of ABCA1 reaching (13 ± 1.15, 10 ± 0.82, 10.88 ± 0.63 ng/mg).

#### Nuclear/cytoplasmic NF-κB and Ror2 protein expression level

**Fig. 8:**
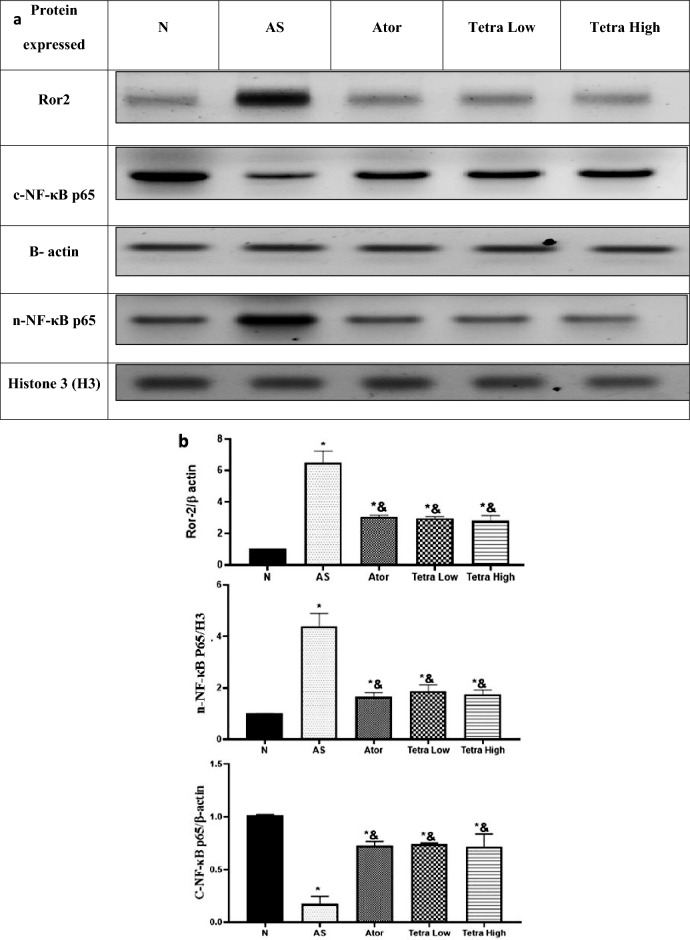
Change in the aortic tissue Ror2 and nuclear/cytoplasm NF-κB expression levels in AS rats induced by vitamin D3/high cholesterol diet (HCD) after administration of atorvastatin at a dose of 20 mg/kg/day, tetrandrine at low or high dose for 22 days. Data represented as mean ± SD of 8 rats. *is significantly different from N. & is significantly different from AS. N: normal control group, AS: Atherosclerotic group, Ator: atorvastatin treated group, Tetra low: tetrandrine treated group at a dose of 18.75 mg/kg, and Tetra high: tetrandrine treated group at a dose of 31.25 mg/kg.

Figure 8, illustrates the change in the aortic expression of Ror2, nuclear and cytoplasmic NF-κB in AS rats after treatment with atorvastatin or tetrandrine in low or high dose as compared to AS untreated rats. The AS rats showed a substantial increase in aortic expression of Ror2, and nuclear NF-κB. While cytoplasmic NF-κB expression was declined in Aorta of AS untreated rats. All treatments given reduced aortic Ror2 and nuclear NF-κB expression while increased cytoplasmic NF-κB expression. There were no significant differences across the treatment groups (Supplementary Figures).

#### The miR-34a expression level

**Fig. 9:**
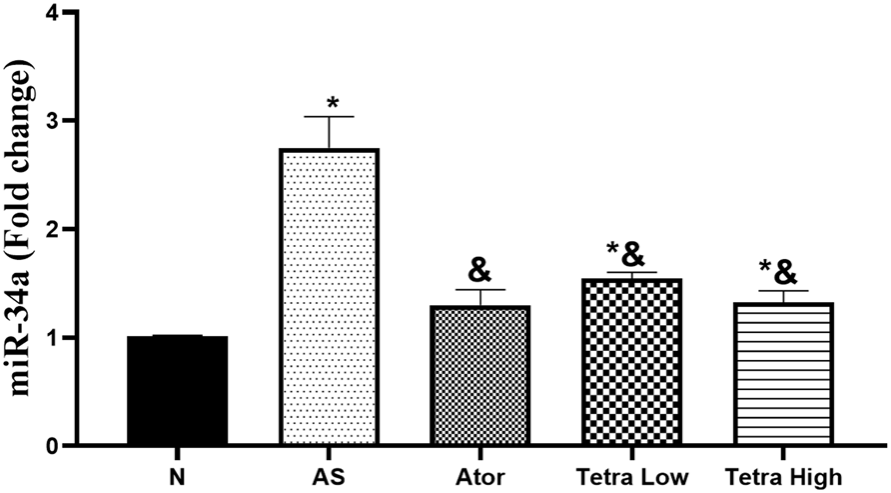
Change in the aortic tissue miR-34a expression level in AS rats induced by vitamin D3/high cholesterol diet (HCD) after administration of atorvastatin at a dose of 20 mg/kg/day, tetrandrine at low or high dose for 22 days. Data represented as mean ± SD of 8 rats. *is significantly different from N. & is significantly different from AS. N: normal control group, AS: Atherosclerotic group, Ator: atorvastatin treated group, Tetra low: tetrandrine treated group at a dose of 18.75 mg/kg, and Tetra high: tetrandrine treated group at a dose of 31.25 mg/kg.

Figure 9, demonstrated that there was a significant elevation in the miR-34a level in AS untreated rats (2.75 ± 0.29-fold change) as compared to normal rats (1.01 ± 0.01-fold change). We observed that there was a significant decline in the level of expression of miR-34a rats in rats treated with atorvastatin (1.30 ± 0.14-fold change). Furthermore, treatment of rats with tetrandrine at low and high doses causes also a significant decrease in the expression level of miR-34a (1.55 ± 0.06, 1.33 ± 0.010-fold change, respectively).

#### Histopathological examination

**Fig. 10:**
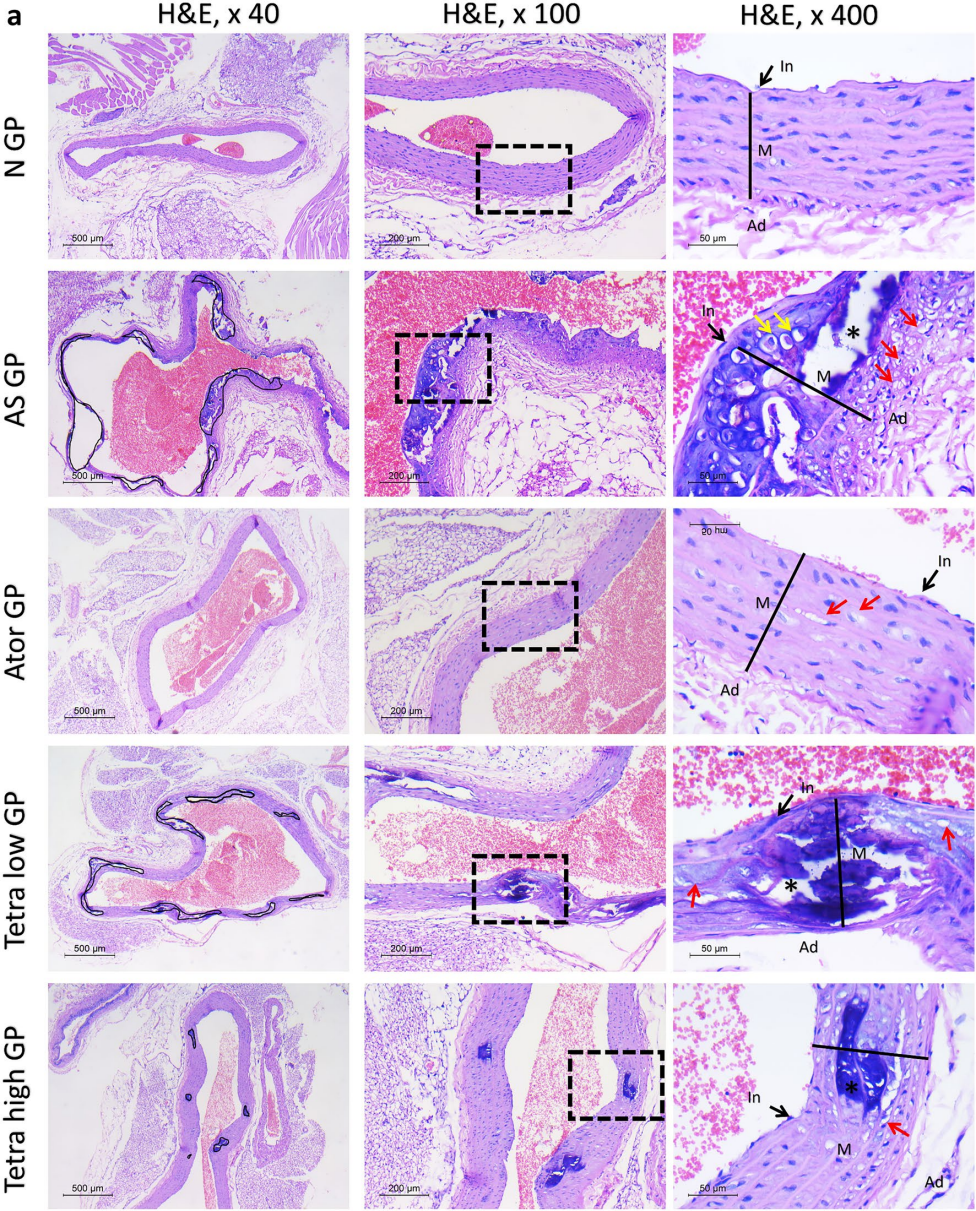

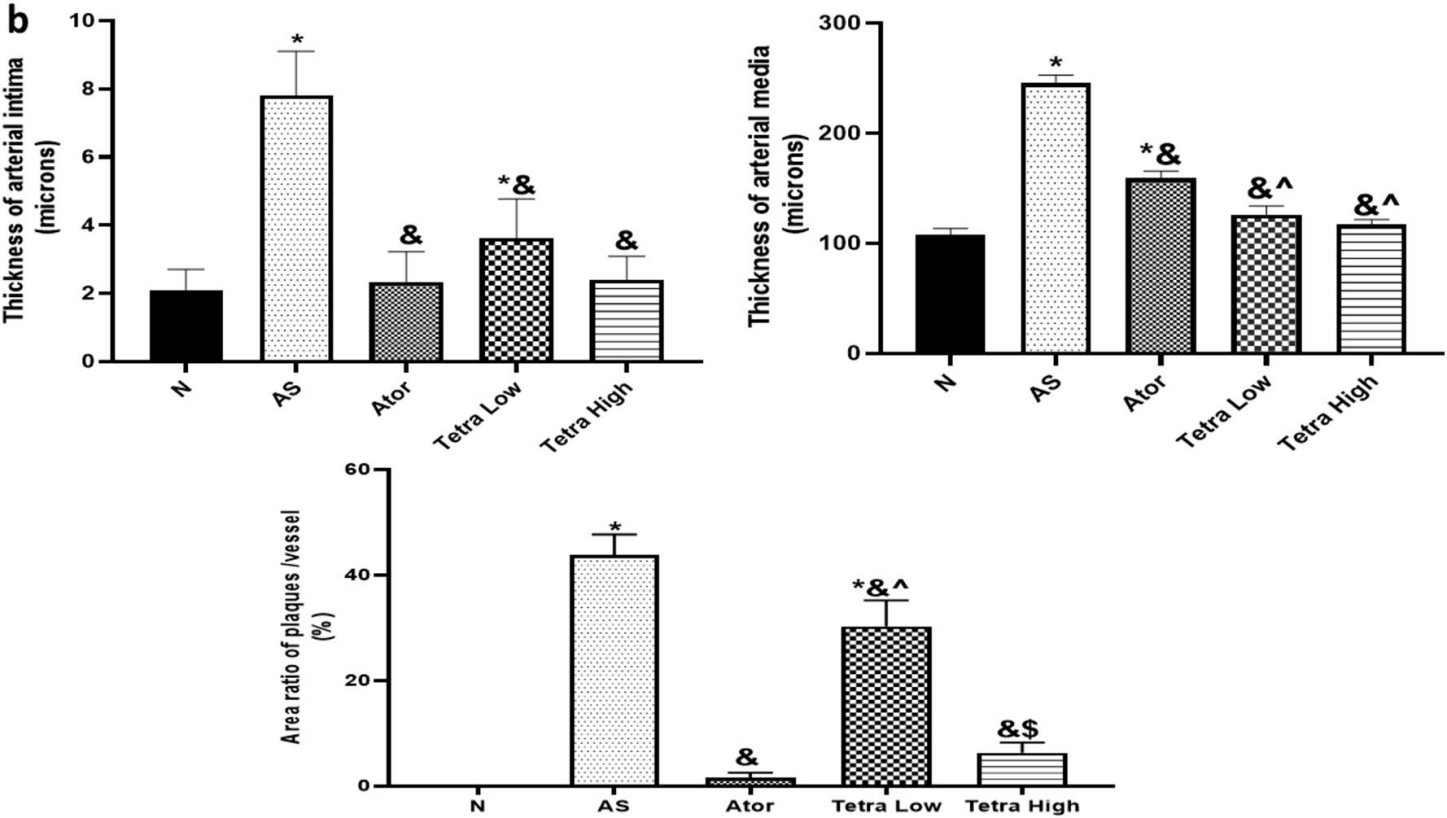
(**a**) Histopathologic assessment of H&E-stained sections of aorta in studied groups: normal group shows normal histology. First column shows atheromas delineated with black line. Note the decline of atheromas percentage in treated rats. Second and third column, highlight vessels wall histology in × 100 and × 400 power. In = intima, M = media, Ad = adventitia. Black line: media thickness. * = calcification. Red arrow = foam cells. (**b**) bar charts for illustrating the thickness of the arterial wall as well as area ratio of plaques/vessel (%) in different experimental groups. Data represented as mean ± SD of 8 rats. *is significantly different from N. & is significantly different from AS. ^ is significantly different from Ator. $ is significantly different from Tetra low. N: normal control group, AS: Atherosclerotic group, Ator: atorvastatin treated group, Tetra low: tetrandrine treated group at a dose of 18.75 mg/kg, and Tetra high: tetrandrine treated group at a dose of 31.25 mg/kg.

Figure 10, illustrates the histopathological changes in the aortic tissues of all experimental groups. The normal groups showed normal histology of aortic vessel. The intima was thin. No plaques or atheromas were seen in the media. While in the atherosclerosis model, large atheromas were frequently seen in the aortic wall. They occupied 43.9% of the total vessel wall. The overlying intima was thickened indicating intimal hyperplasia. The media was markedly expanded with well-formed atheromas. They were formed of a collection of foamy cells and cholesterol cleft with frequently detected central necrosis and dystrophic calcifications. The adventitia was inflamed.

The treated drugs showed evident improvement in the aortic wall histology. The atorvastatin treated rats showed a marked decline in the atheroma number and size (1.62%). On high power examination, intima was thin. Media showed regaining of ordered smooth muscles. Only limited foci of foamy cell aggregation were occasionally seen. Fine dystrophic calcifications were accidentally detected. No inflammation or necrosis was present.

Tetrandrine treatment showed a dose-dependent therapeutic effect as well. In low dose, the atheromas were covered by slightly hyperplastic intima. The tunica media thickness slightly improved. The atheromas percentage dropped to 30.32% of the total area. Lesions were smaller with uncommon necrosis. However, wide areas of dystrophic calcification were seen. In contrast to the high dose tetrandrine which showed better effect. The atheromas were seen in 6.3% of vessel walls examined. The intima was thin and media was arranged in a relatively ordered fashion. Areas of foamy cells and intermittent dystrophic calcifications were rarely detected; however, no necrosis was seen.

#### Special stain analysis for elastic and collagen contents

**Fig. 11:**
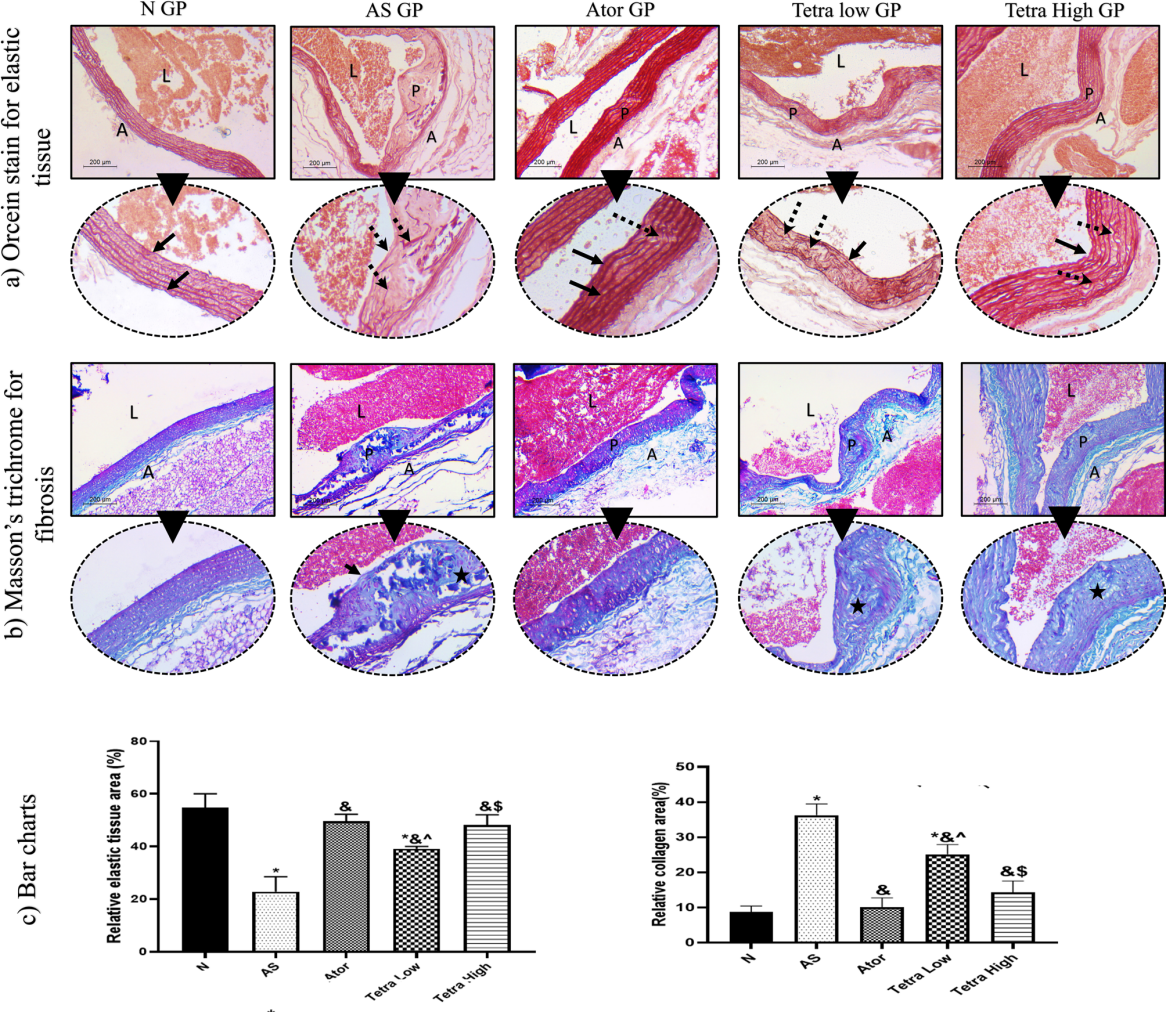
(**a**) Orcein staining of aorta in studied groups to show elastic content within vessel wall. Normal group shows thin vessels wall, on high power intact both inner and outer laminae (black arrows) with parallel elastic laminae of media in-between. In AS group, a well-formed plaque (P) is seen within the wall. The high-power shows disrupted incomplete inner lamina and fragmented elastic laminae of media (dashed arrows). Restoration of architecture and content of elastic laminae was seen in treated group where back arrows point at the intact inner elastic laminae. Meanwhile, dashed arrows point at slightly irregular or focally disrupted laminae. (**b**) Masson trichrome staining of aorta in studied groups to show fibrous content within vessel wall. Normal group shows blue stained adventitia only indicating low fibrous tissue content in media normally. In AS model, a dense blue colored fibrous cap (Black arrow) is seen with wide areas of fibrosis is seen within the plaque (star). Fibrous tissue content was close to normal in Ator treated rats while still areas of fibrosis was seen in low dose Tetra (star) and it declined in high dose (star). (Low power, × 100, high power, × 200)- L = lumen, A = adventitia, P = plaque. (**c**) Bar charts to illustrate the relative elastin tissue and collagen areas (%) in different experimental groups. Data represented as mean ± SD of 8 rats. *is significantly different from N. & is significantly different from AS. ^ is significantly different from Ator. $ is significantly different from Tetra low. N: normal control group, AS: Atherosclerotic group, Ator: atorvastatin treated group, Tetra low: tetrandrine treated group at a dose of 18.75 mg/kg, and Tetra high: tetrandrine treated group at a dose of 31.25 mg/kg.

Orcein stain was able to highlight elastic fibers in the aortic wall by staining it deep red color, Fig. 11. In normal vessels, a complete regular inner elastic lamina was seen as well as outer elastic lamina just before the adventitia. Within the media, parallel complete elastic laminae were detected. This constituted 54.79% of the vessel wall within × 200 power field. In opposite to the atherosclerosis model, the areas of AS atheroma showed evident disruption of inner elastic lamina indicating the presence of fibrous caps. Within the media, the elastic laminae were incomplete, short and interrupted. Elastic tissue constituted less than 22.85% of × 200 fields examined. Near total regains of elastic tissue content and architecture was seen in atorvastatin treated rats (48.6%/ × 200 PF). Partial restoration was seen in low dose tetra (39.18%) which increased to be (48.08%) in high dose.

Masson Trichrome staining was able to stain fibrous tissue blue in color while smooth muscles were stained red, Fig. 11. In the normal aorta, blue stained fibrous tissue was seen mainly in the adventitia and in the collagenous stroma in between smooth muscles of media (8.76%). In the atherosclerosis model, a dense fibrosis cap was seen covering the atheromas. In addition, fibrosis was seen within atheroma (36.28%). The collagen content decreased significantly after treatment. The fibrous caps disappeared. Collagen content was minimal in atorvastatin treated rats (10.10%). While it was 25.13% in low dose tetrandrine and declined to be 14.33% in high dose tetrandrine.

#### Immunohistochemical staining of CD68 and MSA

**Fig. 12:**
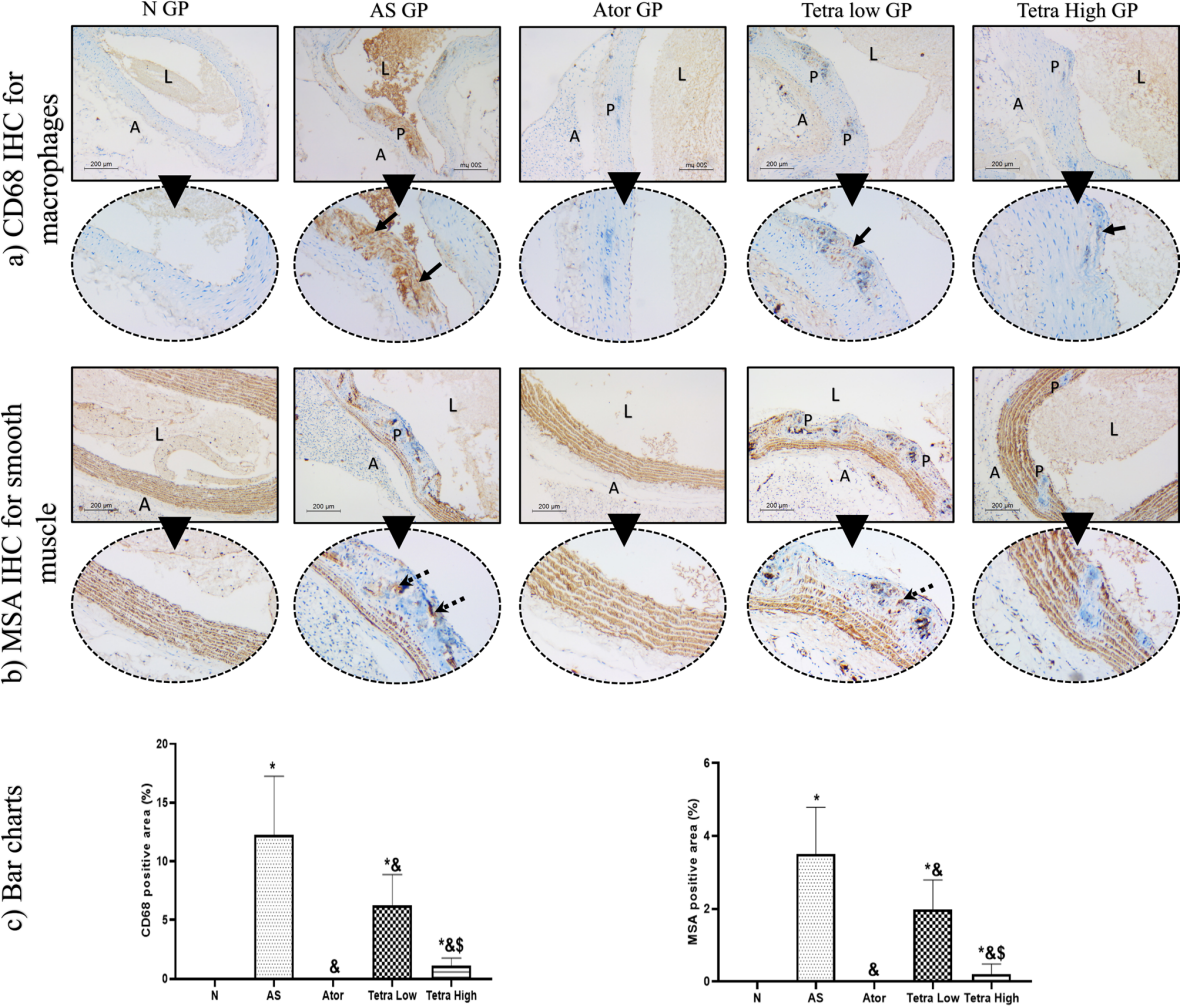
(**a**) Assessment of macrophage content in atheroma by CD68 stain showing negative stain in normal group. wide area of positivity (arrows) is seen in AS group indicating increased macrophage content within plaque. It was negative after Ator treatment. Meanwhile, low dose Tetra showed moderate positivity (arrow) and high dose showed only occasional staining. (**b**) MSA immunostaining highlights smooth muscles in vessel wall. Normal group shows ordinal smooth muscle layers. AS group shows loss of normal muscle layer pattern with scattered positive cells (dashed arrows) within plaques. Normal pattern was totally restored in Ator treated trats. Low dose tetra showed moderate staining (dashed arrows). High dose tetra shows negative staining in plaques with only partial restoration of normal tunica media pattern. (Low power, × 100, high power, × 200)- L = lumen, A adventitia, P = plaque. (**c**) Bar charts illustrating the CD 68 and MSA positive area in different experimental groups. Data represented as mean ± SD of 8 rats. *is significantly different from N. & is significantly different from AS. $ is significantly different from Tetra low. N: normal control group, AS: Atherosclerotic group, Ator: atorvastatin treated group, Tetra low: tetrandrine treated group at a dose of 18.75 mg/kg, and Tetra high: tetrandrine treated group at a dose of 31.25 mg/kg.

The CD68 immunostaining was used to assess the macrophage content in atheromatous plaques in different groups, Fig. 12. Macrophages showed brown cytoplasmic staining and blue nuclei. In the normal group, no macrophages were detected within the vessel wall. Meanwhile, the atherosclerosis model showed large areas of positive brown staining within atheromas. They were detected mainly subintimal indicating macrophage aggregation. The area much decreased in the atorvastatin treated group which showed only focal staining. Tetrandrine treated rats showed dose depended therapeutic effect. The area of CD68 staining was 6.25 and 1.10% respectively in low and high dose tetrandrine.

The MSA staining was used to highlight smooth muscles in atheromas, Fig. 12. In the normal group, brown staining of smooth muscles in media was seen. They showed an ordinal pattern. In the atherosclerosis group, definite distraction and disarrangement of smooth muscles in the media were seen in areas of atheroma. Meanwhile, within the atheromatous plaques, multiple MSA-positive cells were seen indicating smooth muscle migration within the atheromas. The number of positive cells within atheromas and areas decreased after treatment.

## Discussion

Atherosclerosis is one of the leading causes of death globally. Inflammation and oxidative stress play an important role in the pathogenesis of the disease that is exacerbated by a sedentary lifestyle, unhealthy food intake and other diseases such as hyperlipidemia and diabetes^26^. While traditional atherosclerosis treatment choices are successful, many individuals find their side effects intolerable^17^. This study focused on finding an alternative natural remedy to help in the management of the atherosclerosis problem.

Tetrandrine is an alkaloid isolated from the root of *Stephania tetrandra*^27^. Many studies reported its anti-inflammatory, anti-oxidant, anti-cancer, and anti-arrhythmic actions^11–13^. Recently, the invitro ability of tetrandrine to reduce atherosclerosis was explained via its capacity to block the STING-TBK1 pathway and reduce inflammation in macrophages^28^. Based on the obtained data, our study aimed to evaluate the therapeutic potential of tetrandrine, in low and high doses, in vitamin D3/HCD-induced atherosclerosis rat model via regulating miRNA-34a and the Wnt5a/Ror2/NF-κB pathway. Moreover, we wanted to compare the effect of tetrandrine with that of atorvastatin as a reference drug.

In literature, it has been reported that rats were resistant to atherosclerosis development since lipoproteins containing apoB such as (LDL, and VLDL) are of low level. However, HDL is the most prevalent transporter of cholesterol, in rats. So genetically engineered rat models were created^29^. However, in this study, after 9 weeks of feeding on HCD made up of (normal diet (80.3%), animal oil (11%), cholesterol (4.5%), sodium cholate (1.5%), propylthiouracil (0.7%) and refined sugar (4%)) together with IP administration of vitamin D3 (70 U/kg) for 3 continuous days, atherosclerosis was induced in rats. Previously it was mentioned that vitamin D3 was able to accelerate the induction of aortic atherosclerosis in diabetic rats by elevating the gene expression of oxidized-LDL scavenger receptor^30^. Also, it has been reported that vitamin D3 levels aided in the calcification of blood vessels via stimulating both vascular smooth muscle cell osteogenic differentiation and mineralization^31^.

The induction of atherosclerosis in this study was confirmed by histopathological examination of the AS rats' aorta that illustrated the formation of atherosclerotic plaques, aggregation of foamy cells and calcification of aortic wall. Moreover, the level of serum lipids was measured in AS rats which showed that there was a significant increase in the level of total cholesterol, and LDL-C with reduction in the level of HDL-C as compared to normal rats. Unexpectedly, there was a slight increase in the serum TG level that was not significantly different from normal rats. After confirming that induction had occurred, the following treatments (Atorvastatin (20 mg/kg/day), tetrandrine in low (18.75mg/kg/day) and high dose (31.25 mg/kg/day)) were administered for 22 days till the end of the study.

Atherosclerotic plaques result from a chronic systemic elevation in lipids which induces inflammatory as well as oxidative stress conditions that damage the arterial wall^32^. Our results showed that serum inflammatory cytokines (TNF-α, and IL-6) levels were elevated in AS rats as compared to healthy rats. These findings were in line with previous studies^33–35^. Our findings on the serum lipid profile in the same rat atherosclerosis model were validated by earlier research^18,36^.

Oxidative stress condition contributes to atherosclerosis by causing oxidative damage to LDL cholesterol and the endothelial cells lining blood vessels. Plaque development and inflammation are encouraged by this damage. Inflammation also encourages oxidative stress^37^. Our results revealed that there was a significant increase in the liver MDA, and GSSG levels and a decline in the liver total GSH, and reduced GSH levels, in AS untreated rats, as compared to normal healthy rats^35^. These results highlight the interplay between inflammation, hyperlipidemia and oxidative stress in the pathogenesis of atherosclerosis.

Moreover, we found that aortic tissue Wnt5a, Ror2, nuclear-NF-κB and miR-34a expression levels were significantly elevated, while the aortic tissue levels of ABCA1, and cytoplasmic-NF-κB were significantly mitigated in AS untreated rats. The literature highlighted the role of Wnt5a/Ror-2/ABCA1/NF-κB in the pathogenesis of atherosclerosis. It was explained that Wnt5a attaches to Ror2 in the foam cells. This leads to downregulation of ABCA1 expression followed by lipid accumulation and activation of NF-κB provoking inflammation^8,10^. Also, previous studies demonstrated the inverse correlation between miR-34a and ABCA1. They illustrated an increase in the miR-34a expression level in atherosclerosis leads to decrease in the ABCA1 level leading to vicious cycle between hyperlipidemia, oxidative stress and inflammatory conditions^9,38^.

Histopathological assessment of aortic tissue sections stained with H and E showed intimal hyperplasia together with expanded media, collection of foam cells, inflammation and calcification. These findings were similar to other studies done in rat models of atherosclerosis induced by high fat-diet as well as induced by hyperuricemia^39,40^. Another study indicated similar histopathological changes observed in the aorta of AS untreated rats^41^. Orcein and Masson trichrome stains were used to assess the elastic and fibrous tissues, respectively. This assessment showed that the elastic tissue constituted a small percentage of the examined field and a dense fibrous cap covering the atheroma was observed in AS rats' aortic tissues.

Immunohistochemical staining of the CD68 was done to show us the macrophage content in the atheromas indicated by positive brown cytoplasmic stain and blue nuclei. The aorta of AS untreated rats showed macrophage aggregation within the atheromas. The MSA staining was done to assess the smooth muscles in the atheromas indicated by brown stain. The stained aortic sections of AS rats showed disarrangement of smooth muscles in areas of atheroma.

In our study, the rats treated with tetrandrine at low or high doses significantly reduced the serum levels of TNF-α and IL-6 as compared to AS group. There was no significant difference between tetrandrine in both tested doses and the atorvastatin treated group. The anti-inflammatory effect of tetrandrine had been reported previously in rheumatoid arthritis mice model^42^. Also, it was proved that tetrandrine decreased the expression of proinflammatory mediators via inhibition of NF-κB activation in murine BV2 microglial cells and complete Freund's adjuvant (CFA)-induced arthritis rat^15,16,43^.

Our results revealed that treatment with atorvastatin and high dose tetrandrine gave the same inhibitory effect on the measured serum lipids. Their effect was superior to that of low dose tetrandrine. The outcomes of our study were consistent with other studies, illustrating the ability of tetrandrine to reduce the levels of TC, and LDL while increasing the levels of HDL in the arterial blood of rats suffering from myocardial infarction^44^.

Tetrandrine therapy at both tested dosages decreased the amounts of GSSG and MDA in the liver as well as increased total and decreased GSH. Such antioxidant effect of tetrandrine was comparable to that of atorvastatin. The anti-oxidant activity of tetrandrine was previously shown in traumatic brain damage in mice, and ischemia/reperfusion induced neuronal damage in the subacute phase via declining MDA and provoking GSH levels^45,46^. Also, tetrandrine's capacity to counteract oxidative stress to attenuate tertbutyl hydrogen peroxide (TBHP)-induced nucleus pulposus (NP) cell injury was illustrated previously^47^.

The ABCA1, and cytoplasmic NF-κB aortic tissue levels were provoked by treatment with tetrandrine in both doses. Nevertheless, tetrandrine administration at both dosages resulted in a decrease in the levels of Wnt5a, Ror-2, nuclear-NF-κB, and miR-34a in aortic tissue. This effect observed in the previously described metrics was comparable to what the atorvastatin group experienced. Prior research showed that tetrandrine capacity to suppress the Wnt signaling pathway in human colorectal cancer and osteoarthritis in rabbits^15,16,48^. As was previously noted, tetrandrine anti-inflammatory impact resulted from its suppression of NF-κB, in collagen-induced arthritis in mice and complete Freund's adjuvant (CFA)-induced arthritis in rats^43,49^.

Moreover, treatment of AS rats with atorvastatin and tetrandrine showed dose dependent effect on reverting the histopathological changes, seen in the AS aortic tissues, such as decreased % of the atheroma of the total vessel wall reaching (1.62, 30.31, 6.3%), respectively as compared to that of AS group (43.9%). Investigating the orcein stained aortic sections, near total regain of the elastic tissue was observed in the atorvastatin treated group, partial restoration in the elastic tissue was detected in the tetrandrine (low dose) treated group and this was increased with tetrandrine (high dose). In the Masson Trichome-stained aortic sections, there was a significant decline in the collagen content mostly in atorvastatin treated rats followed by high dose tetrandrine, then the low dose. Also, various given treatments reduced the macrophage content and smooth muscle migration in the atheroma with the most significant effect observed in atorvastatin group which wasn’t significantly different from high dose tetrandrine.

## Conclusion

Tetrandrine possess anti-inflammatory and anti-oxidant properties which were illustrated by its capability in reducing the inflammatory cytokines serum levels, as well as mitigating the liver MDA, GSSG and elevating the total and reduced GSH levels. Moreover, it was capable of reducing the aortic levels of Wnt5a/Ror-2 and miR-34a, subsequently increasing ABCA1 level. This leads to a decrease in lipid accumulation, in the aortic wall, and a decline in the activation of the NF-κB, thus reducing inflammation. This highlights the ability of tetrandrine to ameliorate atherosclerosis in rats via modulating miR-34a as well as Wnt5a/Ror-2/ABCA1/ NF-κB pathway. Accordingly, the current work advocates the use of tetrandrine as a promising tool in management of atherosclerosis. Our recommendation is to conduct a study for evaluating the protective efficacy and safety of tetrandrine in atherosclerosis in rats.

## Supplementary Information

Supplementary Figures.

## Data Availability

All data generated or analyzed during this study are included in this published article.

## Abbreviations

AS: Atherosclerosis
CVDs: Cardiovascular diseases
LDL: Low-density lipoproteins
WNT: Wingless
Ror2: Receptor tyrosine kinase-like orphan receptor 2
ABCA1: Adenosine triphosphate (ATP)-binding cassette transporter A1
NF-κB: Nuclear factor kappa B
TNF-α: Tumor necrosis factor-alpha
IL-6: Interleukin-6
HCD: High cholesterol diet
TG: Total triglycerides
TC: Total cholesterol
HDL: High-density lipoprotein
AI: Atherogenic index
MDA: Malondialdehyde
SDS-PAGE: Sodium dodecyl sulfate–polyacrylamide gels
PVDF: Polyvinylidene fluoride membrane
(H3): Histone
H&E: Hematoxylin and eosin
N: Normal control group
AS: Atherosclerotic group
Ator: Atorvastatin treated group
Tetra low: Tetrandrine treated group at a dose of 18.75 mg/kg
Tetra high: Tetrandrine treated group at a dose of 31.25 mg/kg.
CFA: Complete Freund’s adjuvant
TBHP: Tert butyl hydrogen peroxide
NP: Nucleus pulposus
