# Supplementary figures and images for: Tetrandrine ameliorated atherosclerosis in vitamin D3/high cholesterol diet-challenged rats via modulation of miR-34a and Wnt5a/Ror2/ABCA1/NF-kB trajectory
Source: Sci Rep. 2024 Sep 12;14:21371. doi: 10.1038/s41598-024-70872-y (PMC11393063; doi:10.1038/s41598-024-70872-y)

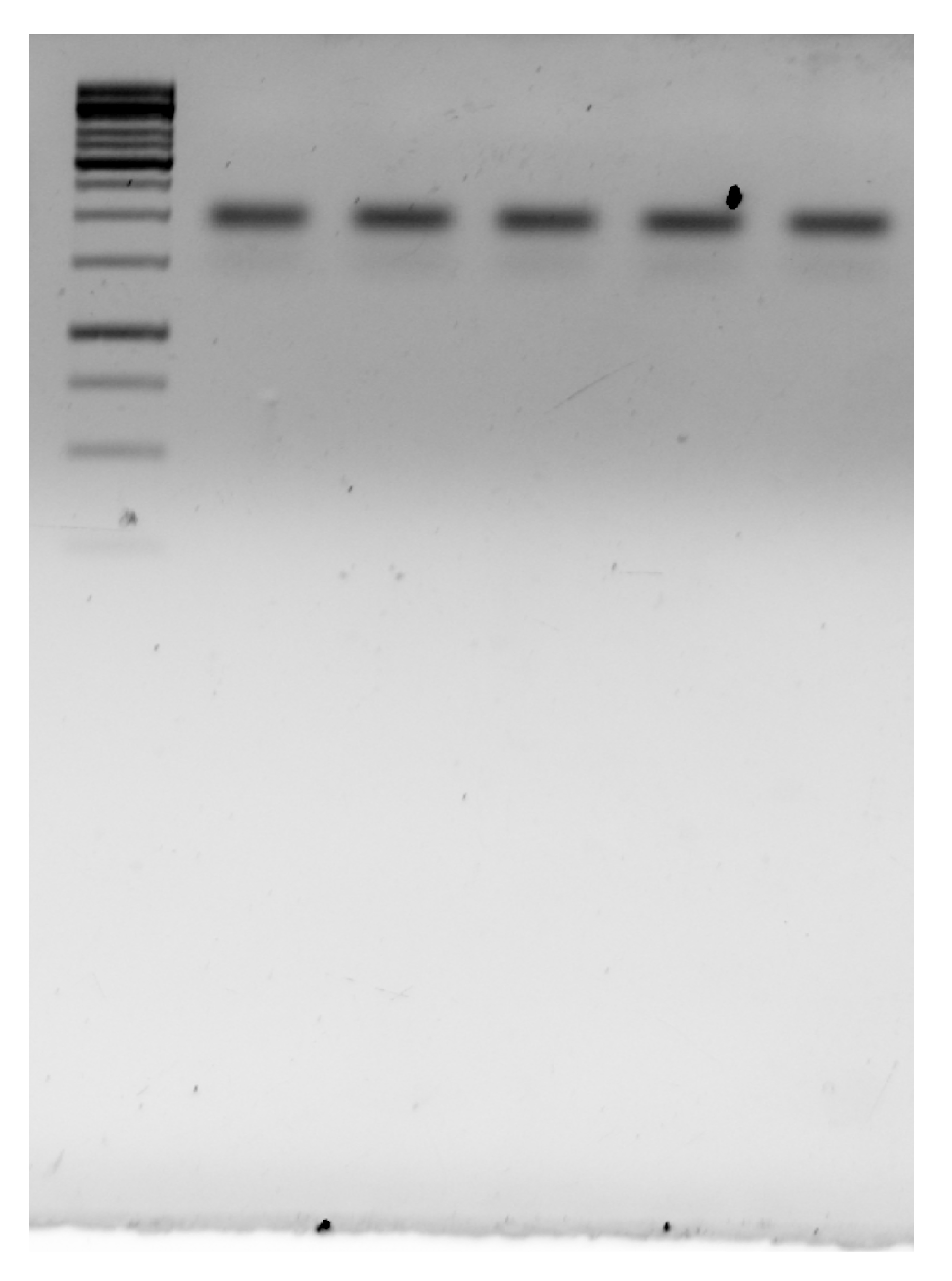

Supplement: Supplementary file 1 — Supplementary Figures. [file 41598_2024_70872_MOESM1_ESM.zip › 41598_2024_70872_MOESM1_ESM/beta actin.jpg]

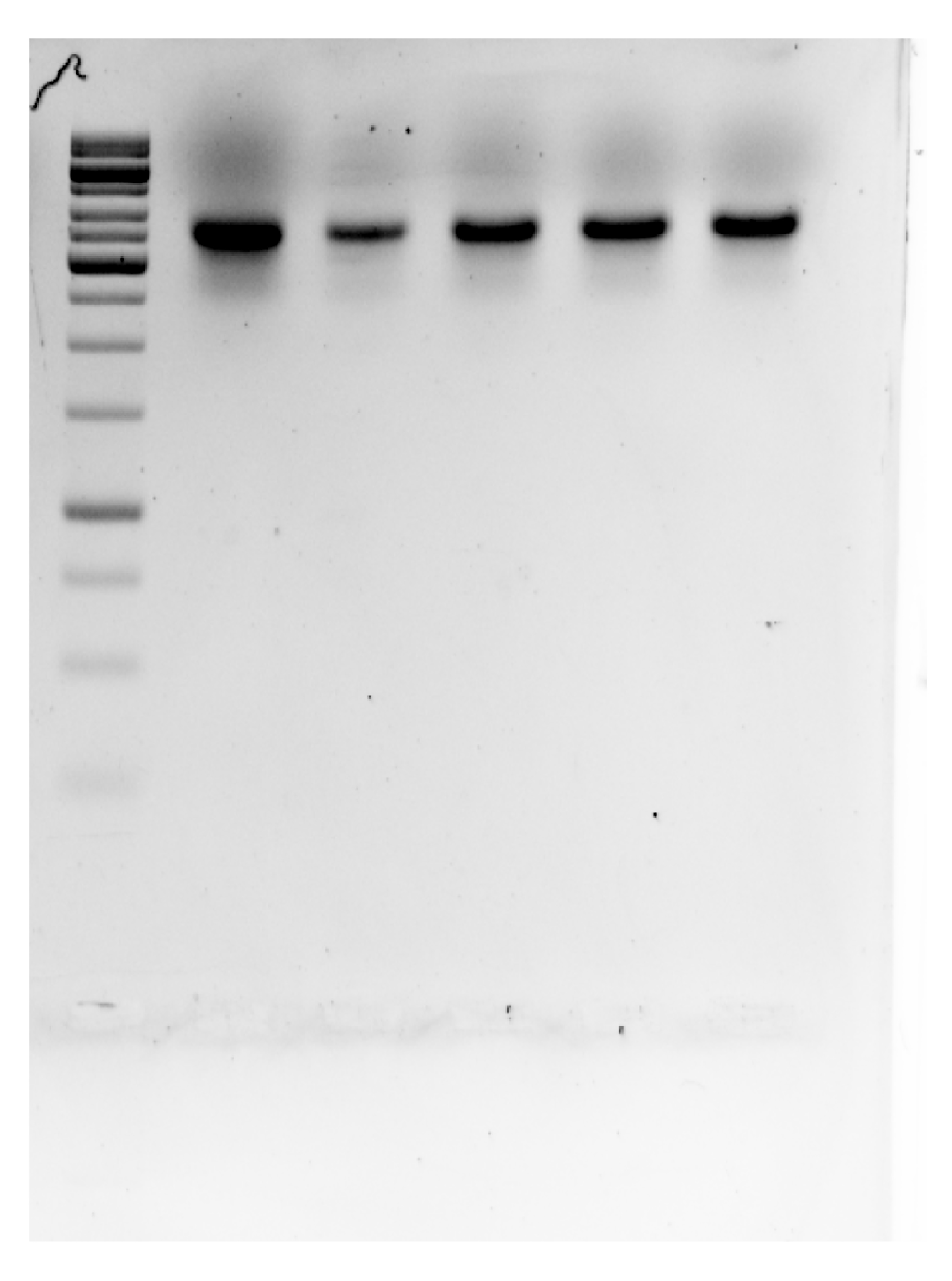

Supplement: Supplementary file 1 — Supplementary Figures. [file 41598_2024_70872_MOESM1_ESM.zip › 41598_2024_70872_MOESM1_ESM/c NFKB.jpg]

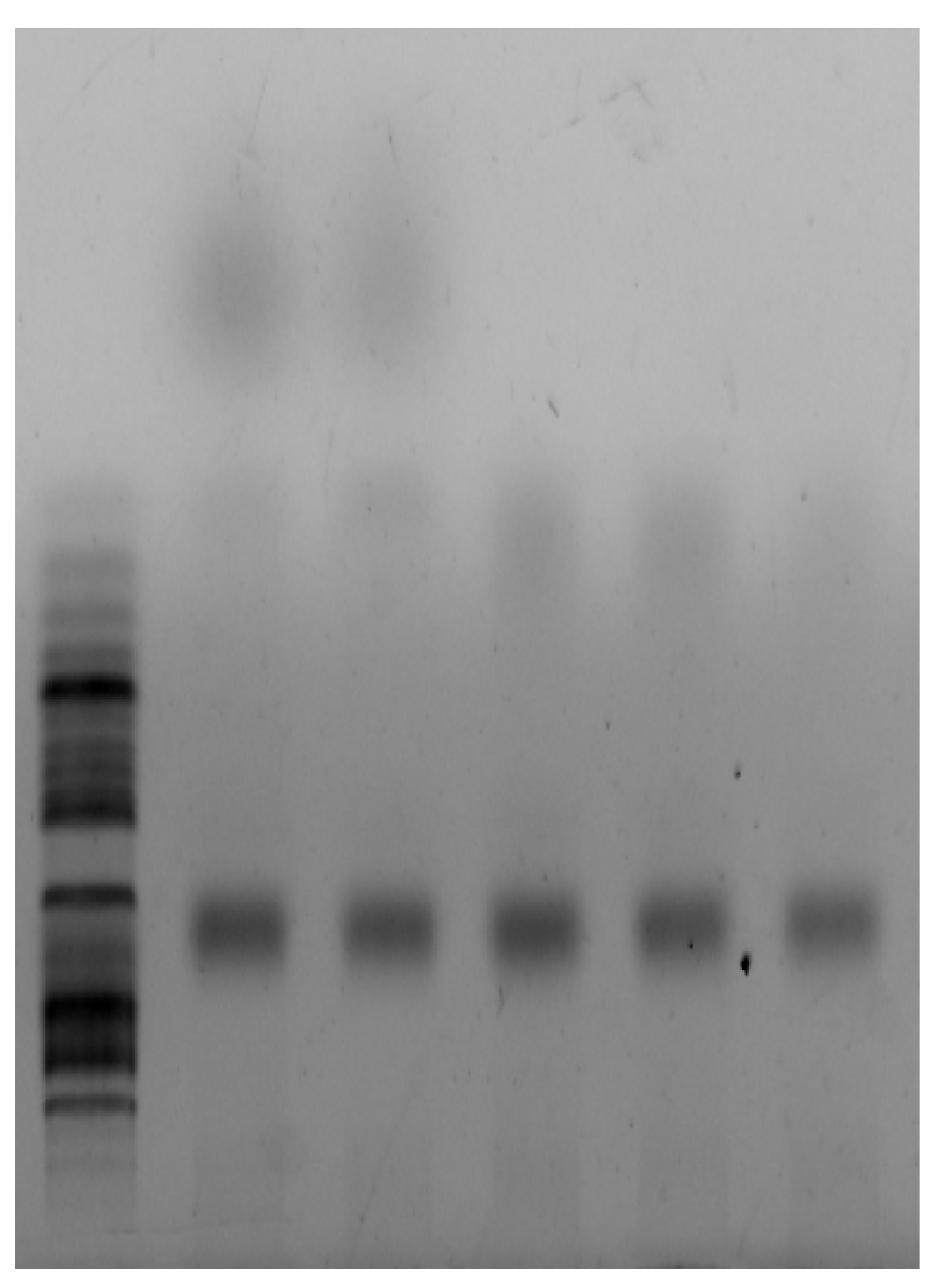

Supplement: Supplementary file 1 — Supplementary Figures. [file 41598_2024_70872_MOESM1_ESM.zip › 41598_2024_70872_MOESM1_ESM/H3.jpg]

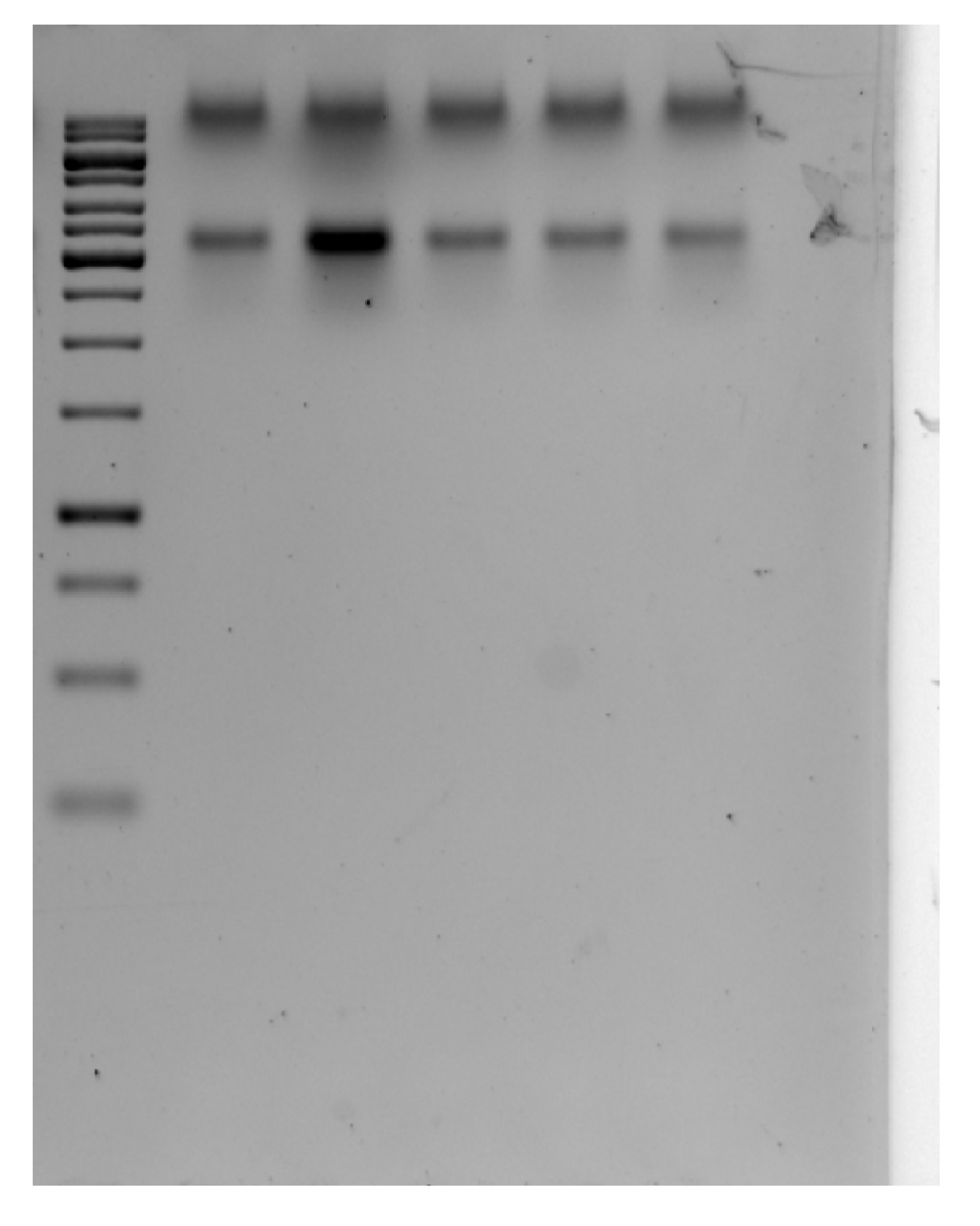

Supplement: Supplementary file 1 — Supplementary Figures. [file 41598_2024_70872_MOESM1_ESM.zip › 41598_2024_70872_MOESM1_ESM/N NFKB.jpg]

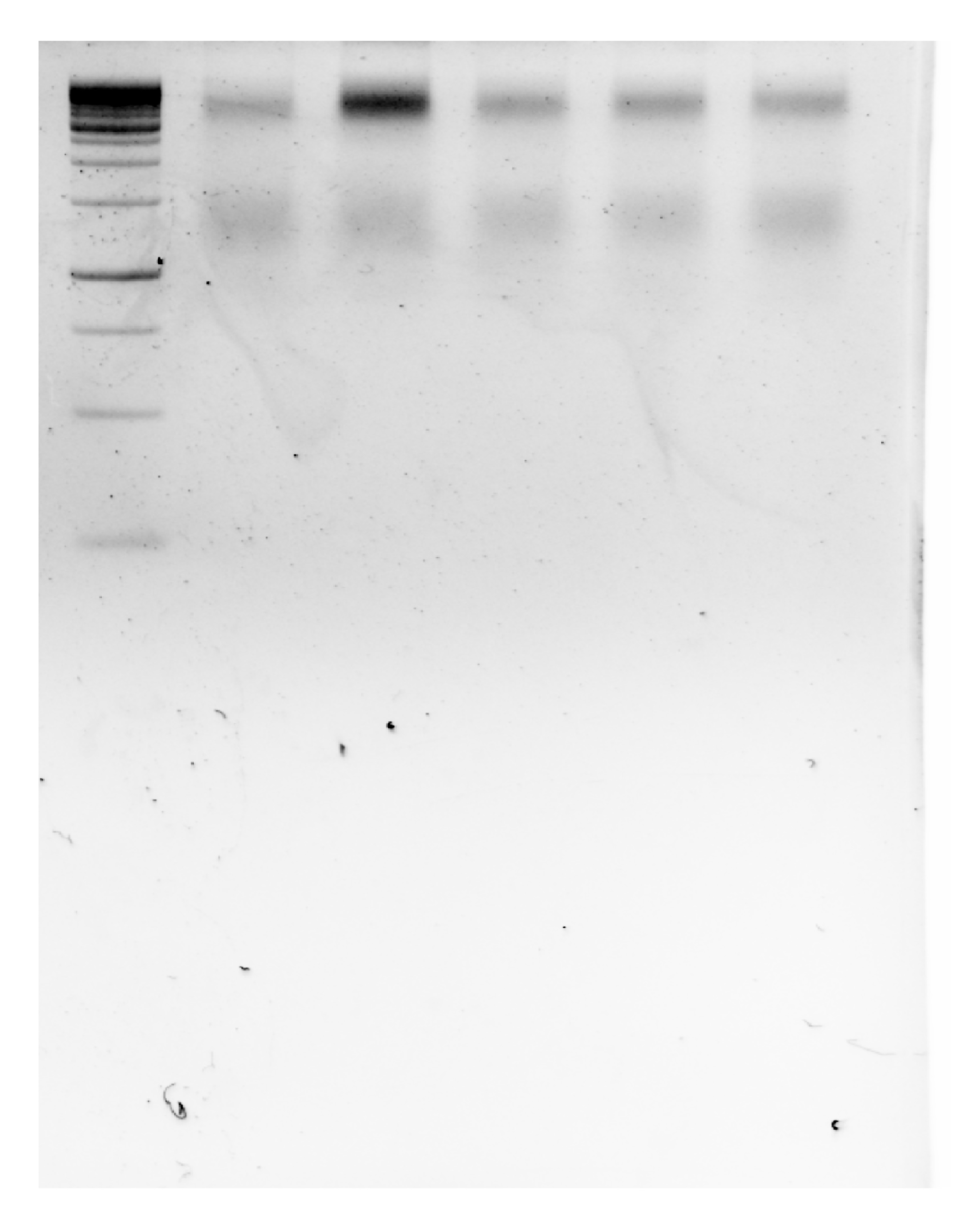

Supplement: Supplementary file 1 — Supplementary Figures. [file 41598_2024_70872_MOESM1_ESM.zip › 41598_2024_70872_MOESM1_ESM/ROR.jpg]
